# In-Vehicle Feedback With or Without Parent Communication Training and Teenage Driving Behaviors: A Randomized Clinical Trial

**DOI:** 10.1001/jamanetworkopen.2026.8631

**Published:** 2026-04-24

**Authors:** Jingzhen Yang, Ying Zhang, Enas Alshaikh, Hannah Schneider, Archana Kaur, Dominique M. Rose, Priyanka Sridharan, Armita Kar, Kele Ding, Yang Wang, Xueyuan Ren, Miao Yu, Lisa Roth, Cara Hamann, Elizabeth E. O’Neal, Motao Zhu, Corinne Peek-Asa

**Affiliations:** 1 Center for Injury Research and Policy at the Abigail Wexner Research Institute, Nationwide Children’s Hospital, Columbus, Ohio; 2 Department of Pediatrics, The Ohio State University, Columbus; 3 Department of Biostatistics, University of Nebraska Medical Center, Omaha; 4 Department of Geography and Geoinformation Science, George Mason University, Fairfax, Virginia; 5 Health Education and Promotion Program, School of Health Sciences, Kent State University, Kent, Ohio; 6 Department of Computer Science and Engineering, The Ohio State University, Columbus; 7 University of Iowa Injury Prevention Research Center, Iowa City; 8 Department of Epidemiology, University of Iowa, Iowa City; 9 Department of Community and Behavioral Health, University of Iowa, Iowa City; 10 Office of Research Affairs, University of California at San Diego

## Abstract

**Importance:**

Motor vehicle collisions are a leading cause of death among teenagers. Effective strategies to reduce risky driving, especially for teenage drivers cited for traffic violations, are critical yet underdeveloped.

**Objective:**

To evaluate the effectiveness of ProjectDRIVE, an in-vehicle and smartphone-based driving feedback intervention combined with parent communication training, in reducing risky driving events and unsafe driving behaviors among teenagers with traffic violations.

**Design, Setting, and Participants:**

This 3-arm, parallel randomized clinical trial enrolled 240 parent-teenager dyads, randomized evenly (80 per arm) to the control arm (device installed without feedback), the driving feedback only arm, or the driving feedback plus parent training arm. Enrollment occurred from September 28, 2020, to June 30, 2024, with 6 months of follow-up. Participants included teenagers aged 16 to 17 years with an intermediate license and a moving violation, and their parent or guardian, enrolled from 6 juvenile traffic courts across Ohio.

**Interventions:**

Intervention teenagers received real-time, in-vehicle and smartphone-based feedback, as well as biweekly emailed reports. Parents in the driving feedback plus parent training arm accessed their teenager’s driving reports, completed virtual communication training, and received an online guide for discussing safe driving.

**Main Outcomes and Measures:**

Intent-to-treat analyses assessed intervention effects on the incidence rate of risky driving events per 1000 miles and the proportion of miles driven involving unsafe driving behaviors, measured using telematics.

**Results:**

Among 240 parent-teenager dyads (teenager mean [SD] age, 16.7 [0.5] years; 123 female teenagers [51.3%]), teenagers completed 160 095 trips. Compared with control, driving feedback plus parent training significantly reduced risky driving event incidence (adjusted incidence rate ratio [AIRR], 0.68; 97.5% CI, 0.51-0.90) and the proportion of miles driven while speeding (adjusted exponentiated β coefficient, 0.54; 97.5% CI, 0.47-0.68). Driving feedback alone did not significantly reduce risky driving but did reduce miles driven while speeding (adjusted exponentiated β coefficient, 0.64; 97.5% CI, 0.54-0.79). Male teenagers exhibited higher rates of risky driving compared with female teenagers, including hard braking (AIRR, 1.39; 95% CI, 1.11-1.73) and sudden acceleration (AIRR, 2.13; 95% CI, 1.42-3.19), as well as greater proportions of miles driven while speeding.

**Conclusions and Relevance:**

Combining driving feedback with parent communication training reduced risky driving among teenagers with traffic violations. Continued parental engagement after licensure, especially after traffic violations, might be key to reinforcing driving safety among teenagers.

**Trial Registration:**

ClinicalTrials.gov Identifier: NCT04317664

## Key Points

**Question:**

Is in-vehicle and smartphone-based driving feedback, alone or combined with parent training, effective in reducing risky driving among teenagers with traffic violations?

**Findings:**

In a randomized clinical trial of 240 parent-teenager dyads after a teenager (aged 16-17 years) traffic violation, combining in-vehicle and smartphone-based driving feedback with parent training significantly reduced the incidence rate of risky driving events and the proportion of miles driven while speeding. Feedback alone did not significantly reduce risky driving but did reduce miles driven while speeding.

**Meaning:**

This study suggests that sustained parental involvement, supported by in-vehicle technology, is important for reinforcing safe driving as teenagers transition to independent driving.

## Teaser

This 3-arm, parallel randomized clinical trial assesses whether in-vehicle and smartphone-based driving feedback, alone or combined with parent training, is effective in reducing risky driving among teenagers with traffic violations.

## Introduction

Despite persistent efforts to reduce crashes among novice teenager drivers, motor vehicle collisions remain a leading cause of death and disability for teenagers and impose substantial burdens on other road users.^1,2,3,4^ Most interventions focus on the early learning phase and the transition to independent driving.^5,6,7,8,9^ Few are designed specifically for teenage drivers after licensure, particularly those with traffic violations.^10,11,12,13^

Parental involvement plays a vital role in teaching, supervising, and reinforcing safe driving habits in teenagers.^6,7,8,14,15,16,17,18,19^ Once teenagers begin driving independently, parental engagement often decreases, even as teenagers continue to develop their skills.^14,20^ Most parent-focused interventions are implemented broadly across teenagers at all risk levels,^8,9,14,18,19,20^ whereas programs tailored to teenagers with traffic violations are scarce,^21,22,23,24^ although some have reduced repeat offenses.^21^ Expanding parental engagement strategies that specifically support teenagers at high risk and incorporate tiered risk approaches beyond general interventions is crucial for reducing teenage driver crash risks.^25^

Advancements in in-vehicle feedback technology and smartphone-based apps offer opportunities to improve teenage driving safety by providing real-time feedback and behavior reports to both teenagers and parents.^26,27,28,29,30,31^ Research on how these tools can effectively engage parents in shaping teenagers’ driving behaviors remains limited. The period immediately after a traffic violation represents a critical window for promoting safer driving, especially with parental support. Few interventions have specifically targeted teenagers receiving citations, despite this group’s elevated crash risk due to ongoing risk-taking and unsafe driving behaviors.^32^ Developing and evaluating interventions that combine in-vehicle feedback technology with parent training for these teenagers could address critical gaps in both research and prevention.^14,17,33^

## Methods

### Study Design

This randomized clinical trial evaluated the effectiveness of ProjectDRIVE, an in-vehicle and smartphone-based driving feedback intervention combined with parent communication training, in improving safe driving behaviors among teenage drivers with traffic violations (trial protocol in Supplement 1).^34^ We assessed whether driving feedback, alone or combined with parent training, reduced the incidence of risky driving events per 1000 miles driven and the proportion of miles driven involving unsafe driving behaviors. The study was registered on ClinicalTrials.gov (NCT04317664) on March 19, 2020. The first participant was enrolled on September 28, 2020, and recruitment was completed on June 30, 2024. The primary completion date was December 31, 2024, and the study completion date was December 31, 2025. The institutional review boards provided ethical approval at the participating institutions as a single institutional review board at the lead institution (Nationwide Children’s Hospital). The protocol, informed consent forms, and all other relevant trial documents have also received ethical approval. The written informed consent and assent were obtained from the parents and/or legal guardians and the teenager participants, respectively. Throughout the study, we met with the Data Safety Monitoring Committee to ensure participant safety. No adverse events were identified. The trial follows the Standard Protocol Items: Recommendations for Interventional Trials (SPIRIT) reporting guideline and the Consolidated Standards of Reporting Trials (CONSORT)^35^ reporting guideline, the Declaration of Helsinki,^36^ the conditions and principles of Good Clinical Practice, the study protocol, and the applicable local regulatory requirements and laws.

This randomized clinical trial was a 3-arm, parallel trial involving 1 control arm and 2 intervention arms: driving feedback only and driving feedback plus parent training. The study enrolled teenage drivers cited for traffic violations and their parent or legal guardian (hereafter, *parent*) from 6 juvenile traffic courts (Franklin, Delaware, Cuyahoga, Summit, Greene, and Warren counties) across Ohio. A detailed study protocol has been published elsewhere before the conclusion of study recruitment.^34^

### Study Participants

**Figure 1.**
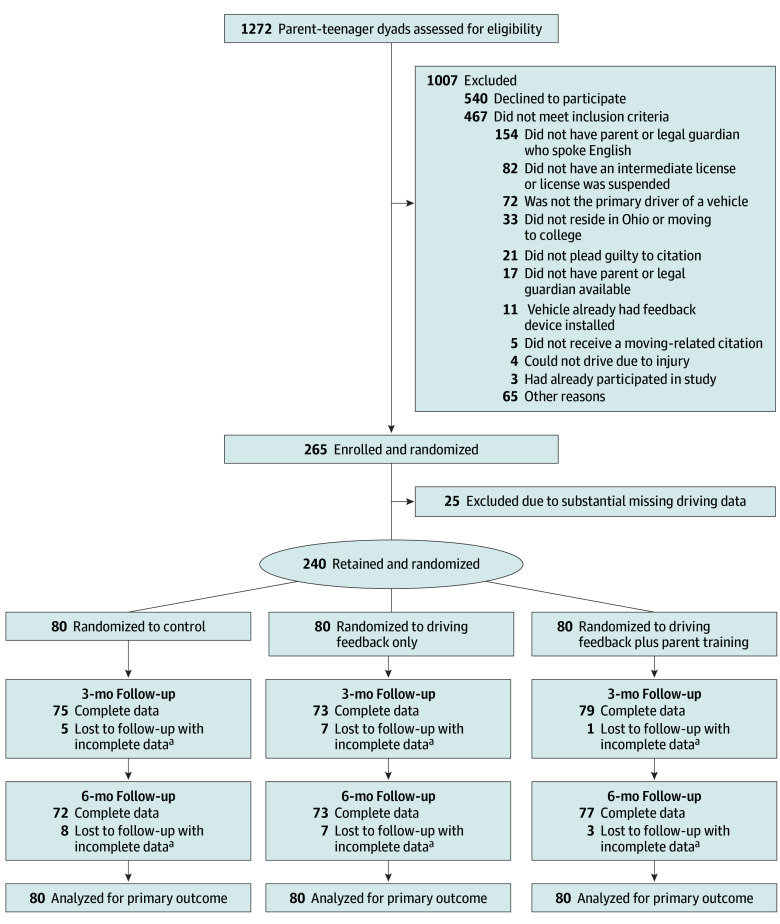
ProjectDRIVE Flow Diagram ^a^Participants with incomplete data (n = 18) were lost to follow-up but had substantial driving data to be included in analysis. Lost to follow-up reasons included loss of communication (n = 10), personal reasons (n = 4), loss of vehicle (n = 3), and feedback device concerns (n = 1).

Participants were 240 parent-teenager dyads consisting of teenage drivers aged 16 or 17 years with an intermediate license who had received a moving violation citation (eg, speeding or failure to yield) and one of their parents. Inclusion criteria were age 16 to 17 years at the time of citation, receipt of a moving violation, a valid intermediate driver’s license, and regular access to a vehicle as the primary driver. Exclusion criteria included inability to drive due to injury, license suspension, or vehicle damage; prior use of an in-vehicle feedback system; inactive driving (eg, <1 hour per week); and non-English speaking.^34^ Between September 2020 and June 2024, 265 parent-teenager dyads were enrolled and randomized; 25 were excluded due to substantial missing driving data, yielding a final analytic sample of 240 dyads (80 per arm) (Figure 1).

### Randomization

We used stratified block randomization by sex (male vs female) to account for known sex differences in traffic violation risk.^5^ Within sex-matched blocks of 3, teenagers were randomized to 1 of 3 groups using a sequence generated by SAS, version 9.4 (SAS Institute Inc). Data analysts were blinded to group assignments.

### Study Procedures

Parent-teenager dyads were recruited in person from 6 county juvenile traffic courts after required hearings, emphasizing rehabilitation rather than punishment.^37^ We posted study flyers at these courts and distributed information sheets to parent-teenager dyads during their court appearances. On recruitment days, staff briefly introduced the study during the judge’s or magistrate’s opening remarks, emphasizing voluntary participation. Interested dyads met with on-site staff after their hearings to receive details, ask questions, complete eligibility screening, and schedule a virtual follow-up meeting. During this meeting, written informed consent was obtained from parents and assent from teenagers via REDCap. Only dyads who provided consent or assent were enrolled. Judges were blinded to the identities of enrolled dyads,^34^ which were randomized within sex-matched blocks to 1 of 3 study arms:

#### Control

For the control group, the Azūga device and app (Azūga, a Bridgestone Company) were installed with feedback features disabled. Dyads received no driving feedback or parent communication training.

#### Driving Feedback Only

For the group receiving driving feedback only, the device and app were installed with feedback features enabled.^38^ Teenagers received real-time feedback and biweekly email reports; parents received no feedback or training.

#### Driving Feedback Plus Parent Training

For the group receiving driving feedback plus parent training, the device and app were installed with feedback enabled. Parents received virtual communication training from a traffic safety specialist, a booster session, an online guide, and access to their teenager’s biweekly reports.

All 3 arms were scheduled for device installation within 2 weeks of enrollment, either in person or virtually, after receiving the device by mail. Research staff guided teenagers through installing the device and app, provided use instructions, answered questions, and shared contact information. Parents assigned to driving feedback plus parent training were scheduled for a communication training session and a booster session with a traffic safety specialist at 2 weeks and 2 months after enrollment. Driving data were monitored through a web interface. Each dyad participated for 6 months: 3 months of active intervention followed by 3 months of follow-up. Parents and teenagers received compensation for completing baseline, 3-month, and 6-month REDCap surveys assessing parent-teenager communication about driving safety.^34^ Regardless of arm assignment, parents received up to $180 and teenagers received up to $240 via debit cards designed for clinical research. Parents in the driving feedback plus parent training arm received an additional $20 for completing surveys after their individualized communication training.

### Intervention Components

#### In-Vehicle Driving Feedback Technology

The device and app worked together to detect risky driving events and behaviors, deliver feedback, and record trip routes. Two types of feedback were provided: real-time feedback and biweekly reports.

#### Real-Time Feedback

Intervention teenagers received 3 types of real-time driving feedback from the device and app. They were (1) audio alerts triggered when vehicle movements exceeded set *g*-force thresholds (hard braking ≤−0.45*g*; sudden acceleration >0.35*g*)^39,40^ or when driving over 75 miles per hour (to convert to kilometers, multiply by 1.6); (2) push notifications delivered at the end of trip if the teenager drove more than 10 miles per hour over the posted speed limit (determined using Google Maps Roads API and OpenStreetMap)^38^; and (3) posttrip summaries showing detailed cumulative driving data, allowing teenagers to review their routes, locations, and the timing of risky driving events and behaviors.

#### Biweekly Reports

Customized reports were delivered every 2 weeks via web, app, email, and text message. These reports included a summary of trips, risky driving events, the teenager’s driving score compared with all study participants, and tailored motivational messages encouraging safer driving, with specific suggestions based on the teenager’s performance.

### Parent Training

Parents in the driving feedback plus parent training arm received guidance to improve communication with their teenagers on safe driving topics. The training was based on the Steering Teens Safe program developed by our team.^8,15,16,17^

#### Virtual Communication Training

Within 2 weeks of enrollment, parents received a 50-minute virtual training from a traffic safety specialist, focusing on motivational interviewing techniques: open-ended questions, affirmations, reflective listening, and summarizing (OARS) for effective communication.^41,42,43^ Parents practiced these strategies through role play and received biweekly email reminders to review their teenager’s driving data and practice communication strategies. A booster session, which included a review of parent-teenager conversations, was held 2 months after enrollment.^34^

#### Online Communication Guide

After the virtual training, parents received an online guide with motivational interviewing demonstration videos and 26 safe driving lessons. These lessons covered basic safety principles, important skills, special driving situations, and setting driving restrictions. Parents completed 2 to 3 lessons per week during the 3-month intervention period, using their teenager’s biweekly driving summary report to guide conversations.^34^

### Primary Outcomes

The incidence rate of risky driving events per 1000 miles was calculated as the number of risky driving events per trip divided by miles driven, multiplied by 1000, over the 6-month study period. The device and app continuously recorded telematics data every 2 minutes during each trip (from ignition on to ignition off), including miles driven. Risky driving events—kinematic events (hard braking ≤−0.45*g*; sudden acceleration >0.35*g*)^39,40^ and speeding events (driving >10 miles per hour over the posted limit or at excessive speeds; eg, >75 miles per hour)^38^—were automatically detected and counted for each trip.

The proportion of miles driven involving unsafe driving behaviors was defined as miles driven with unsafe behaviors divided by total miles driven per trip, over the 6-month study period. Unsafe driving behaviors included (1) miles driven while speeding recorded via telematics, (2) miles driven without seatbelt use recorded via telematics for selected vehicle model years, and (3) self-reported distracted driving behaviors (eg, hands free and handheld phone use, texting, reading texts, viewing, and posting to social media) in the past 2 weeks, assessed via biweekly surveys.

### Other Variables

Other variables included self-reported teenager and parent demographics, including race and ethnicity (African American or Black, Hispanic or Latino, White, or other [Asian or Asian American, Mexican American, Puerto Rican or other Latin American, and other]) to examine demographic differences, as well as trip-level data (date, time, global positioning system [GPS] location, mileage, and duration). Two prespecified secondary outcomes—parent-teenager communication frequency and quality and teenager traffic violation recidivism—were measured but not analyzed here. Analyses were conducted at the trip level, with miles driven included as an offset in regression models for count outcomes.

### Statistical Analysis

We excluded trips from the first 7 days after device installation to allow for sensor calibration. To ensure a consistent observation window and minimize inclusion of nonparticipant drivers, we excluded trips more than 200 days after installation and trips outside Ohio (n = 1177 [0.7%]). The final analytic sample included 160 095 trips across the control (n = 52 458), driving feedback only (n = 51 082), and driving feedback plus parent training (n = 56 555) arms.

Descriptive analyses summarized participant demographics and teenage driving profiles overall and across study arms. Intent-to-treat analyses assessed intervention effects on 2 primary outcomes, the incidence rate of risky driving events and the proportion of miles driven involving unsafe behaviors, using unadjusted and sex- and age-adjusted models. Because trips were repeated measures with excess zero events, mixed-effects zero-inflated regression models were used. Negative binomial models with miles driven as an offset estimated risky driving event rates, and Beta models estimated proportions of miles driven involving unsafe driving behaviors.^44^

Differences across study arms were tested at α = .025 to account for 2 prespecified coprimary end points (trial protocol in Supplement 1),^34^ maintaining an overall type I error rate of .05. The estimates of incidence rate ratios (IRRs) and proportion ratios with their corresponding 97.5% CIs were reported. Arm-by-sex and arm-by-age interactions were not significant and were excluded. Exploratory analyses examined specific risky driving event rates and proportions of miles driven involving specific speeding behaviors and seatbelt nonuse. Because seatbelt nonuse was detectable only in selected vehicle model years (n = 83) and mileage-based denominators were unavailable for self-reported distracted driving, descriptive results are presented in eTables 1 and 2 in Supplement 2. Data analyses were conducted using SAS, version 9.4 (SAS Institute Inc) and R, version 4.4 (glmmTMB package; R Project for Statistical Computing) and completed in December 2025.

## Results

### Participant Demographics

Among 240 participating teenagers, the mean (SD) age was 16.7 (0.5) years, 123 (51.3%) were female, 117 (48.8%) were male, 147 (61.3%) were 17 years of age, 16 (6.7%) were African American or Black, 15 (6.3%) were Hispanic or Latino, 192 (80.0%) were White, 32 (13.3%) were other race, and 19 (7.9%) were other ethnicity (Table 1). Among 240 enrolled parents, the mean (SD) age was 48.9 (6.2) years, 180 (75.0%) were female, 60 (25.0%) were male, 122 (50.8%) were aged 40 to 49 years, 16 (6.7%) were African American or Black, 8 (3.3%) were Hispanic or Latino, 209 (87.1%) were White, 15 (6.3%) were other race, and 16 (6.7%) were other ethnicity, 184 (76.7%) were married, 187 (77.9%) had at least a 4-year college degree, and 41 (17.1%) had a household income below $80 000. Overall, most parent-teenager dyad characteristics were comparable across study arms.

**Table 1.** Parent-Teenager Dyad Demographic and Teenage Driving Profile Characteristics

Characteristic	Participants, No. (%)			
	Overall (N = 240)	Control (n = 80)	Driving feedback only (n = 80)	Driving feedback plus parent training (n = 80)
**Teenager demographic characteristics**				
Age, y				
16	93 (38.8)	31 (38.8)	33 (41.3)	29 (36.3)
17	147 (61.3)	49 (61.3)	47 (58.8)	51 (63.8)
Sex				
Male	117 (48.8)	39 (48.8)	39 (48.8)	39 (48.8)
Female	123 (51.3)	41 (51.3)	41 (51.3)	41 (51.3)
Race				
African American or Black	16 (6.7)	5 (6.3)	7 (8.8)	4 (5.0)
White	192 (80.0)	68 (85.0)	55 (68.8)	69 (86.3)
Other^a^	32 (13.3)	7 (8.8)	18 (22.5)	7 (8.8)
Ethnicity				
Hispanic or Latino	15 (6.3)	5 (6.3)	6 (7.5)	4 (5.0)
Not Hispanic or Latino	206 (85.8)	68 (85.0)	70 (87.5)	68 (85.0)
Other^b^	19 (7.9)	7 (8.8)	4 (5.0)	8 (10.0)
Type of citation				
Speeding related	104 (43.3)	38 (47.5)	37 (46.3)	29 (36.3)
Nonspeeding related	136 (56.7)	42 (52.5)	43 (53.8)	51 (63.7)
Does teenager have his or her own car?				
No	24 (10.0)	11 (13.8)	8 (10.0)	5 (6.3)
Yes	216 (90.0)	69 (86.3)	72 (90.0)	75 (93.8)
**Parent demographic characteristics**				
Age range, y				
≤39	16 (6.7)	2 (2.5)	11 (13.8)	3 (3.8)
40-49	122 (50.8)	38 (47.5)	38 (47.5)	46 (57.5)
≥50	87 (36.3)	33 (41.3)	25 (31.3)	29 (36.3)
Unknown	15 (6.3)	7 (8.8)	6 (7.5)	2 (2.5)
Sex				
Male	60 (25.0)	25 (31.3)	17 (21.3)	18 (22.5)
Female	180 (75.0)	55 (68.8)	63 (78.8)	62 (77.5)
Race				
African American or Black	16 (6.7)	4 (5.0)	7 (8.8)	5 (6.3)
White	209 (87.1)	72 (90.0)	67 (83.8)	70 (87.5)
Other^c^	15 (6.3)	4 (5.0)	6 (7.5)	5 (6.3)
Ethnicity				
Hispanic or Latino	8 (3.3)	2 (2.5)	3 (3.8)	3 (3.8)
Not Hispanic or Latino	216 (90.0)	72 (90.0)	74 (92.5)	71 (88.8)
Other^d^	16 (6.7)	6 (7.5)	3 (3.8)	6 (7.5)
Marital status				
Married	184 (76.7)	68 (85.0)	54 (67.5)	62 (77.5)
Divorced	34 (14.2)	7 (8.8)	16 (20.0)	11 (13.8)
Other^e^	22 (9.2)	5 (6.3)	10 (12.5)	7 (8.8)
Highest level of education				
Some high school or high school graduate	16 (6.7)	4 (5.0)	6 (7.5)	6 (7.5)
Some college	37 (15.4)	12 (15.0)	14 (17.5)	11 (13.8)
4-y College graduate	96 (40.0)	33 (41.3)	28 (35.0)	35 (43.8)
Some graduate school or higher	91 (37.9)	31 (38.8)	32 (40.0)	28 (35.0)
Income level, $				
<80 000	41 (17.1)	6 (7.5)	22 (27.5)	13 (16.3)
≥80 000	185 (77.1)	69 (86.3)	54 (67.5)	62 (77.5)
Refuse to specify	14 (5.8)	5 (6.3)	4 (5.0)	5 (6.3)
**Teenage driving profile characteristics**				
Total trips driven in the study period, No. (%)	160 095 (100)	52 458 (33.0)	51 082 (32.0)	56 555 (35.0)
Miles driven per trip, mean (SD)	6.6 (8.9)	6.4 (8.7)	6.7 (8.9)	6.7 (9.0)
Mean (SD) No. of risky driving events [rate per 1000 miles]^f^	0.6 (0.8) [95.1]	0.7 (0.8) [101.3]	0.7 (0.9) [106.3]	0.5 (0.5) [77.7]
Mean (SD) No. of hard braking events [rate per 1000 miles]^f^	0.3 (0.2) [39.3]	0.3 (0.3) [43.5]	0.3 (0.3) [43.1]	0.2 (0.1) [31.3]
Mean (SD) No. of sudden acceleration events [rate per 1000 miles]^f^	0.2 (0.4) [27.7]	0.2 (0.5) [27.0]	0.2 (0.4) [32.6)	0.2 (0.2) (23.6]
Mean (SD) No. of speeding events >10 miles/h over the posted speed limit [rate per 1000 miles]^f^	0.1 (0.4) [12.4]	0.1 (0.5) [14.9]	0.1 (0.4) [12.4]	0.1 (0.5) [9.8]
Mean (SD) No. of speeding events >75 miles/h, [rate per 1000 miles]^f^	0.1 (0.4) [15.7]	0.1 (0.4) [15.9]	0.1 (0.4) [18.2]	0.1 (0.3) [13.0]
Proportion of miles driven involving speeding, mean (SD)^g^	0.4 (0.1)	0.5 (0.1)	0.4 (0.1)	0.4 (0.1)

^a^ The “other” category for teenager-reported race included the following responses: Asian or Asian American (n = 4), Mexican American (n = 1), Puerto Rican or other Latin American (n = 1), and other (n = 26).

^b^ The “other” category for teenager-reported ethnicity included the following responses: refuse to specify (n = 3) and missing (n = 16).

^c^ The “other” category for parent-reported race included the following responses: Asian or Asian American (n = 5), Mexican American (n = 2), Puerto Rican or other Latin American (n = 2), other (n = 5), and refuse to specify (n = 1).

^d^ The “other” category for parent-reported ethnicity included the following responses: refuse to specify (n = 1) and missing (n = 15).

^e^ The “other” category for parent marital status included the following responses: separated (n = 5), widowed (n = 3), and single (n = 14).

^f^ Rate per 1000 miles = (mean number of events per trip/mean miles driven per trip) × 1000.

^g^ Proportion of miles driven involving speeding among trips with at least 1 speeding event.

### Teenage Driving Profile Characteristics

**Figure 2.**
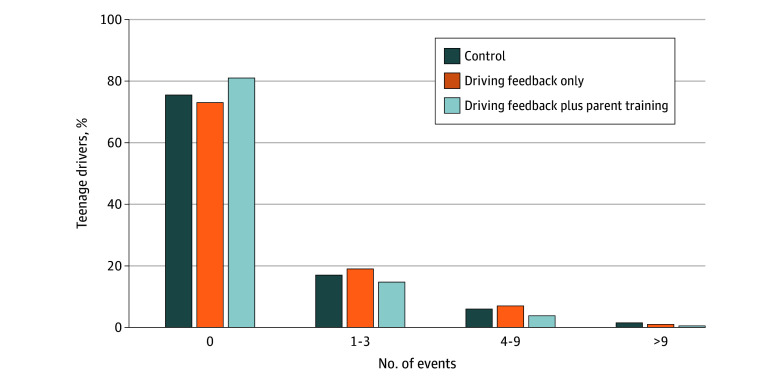
Frequency of Risky Driving Events by Study Arm

Of the 160 095 included trips, 21.9% (n = 35 052) involved at least 1 risky driving event. Teenagers in the driving feedback plus parent training arm had a higher proportion of trips without risky driving events than teenagers in the control arm (81.0% [45 810 of 56 555] vs 75.5% [39 606 of 52 458]) (Figure 2). Teenagers drove a mean (SD) of 6.6 (8.9) miles per trip, with similar distances across study arms (Table 1). The mean (SD) number of risky driving events per trip was 0.6 (0.8), corresponding to 95.1 events per 1000 miles driven. Incidence rates were 101.3 per 1000 miles in the control arm, 106.3 per 1000 miles in the driving feedback only arm, and 77.7 per 1000 miles in driving feedback plus parent training arm. Among trips with at least 1 speeding event, the mean (SD) proportion of miles driven while speeding was 0.4 (0.1), with corresponding mean (SD) values of 0.5 (0.1) in the control arm, 0.4 (0.1) in the driving feedback only arm, and 0.4 (0.1) in the driving feedback plus parent training arm.

### IRRs and Proportion Ratios of Primary Outcomes

The incidence rate of risky driving events per 1000 miles differed significantly across study arms (Table 2). Compared with the control arm, teenagers in the driving feedback plus parent training arm had significantly lower incidence rates in both unadjusted (IRR, 0.70; 97.5% CI, 0.52-0.94) and adjusted analyses (adjusted IRR [AIRR], 0.68; 97.5% CI, 0.51-0.90); no significant effects were observed in the driving feedback only arm. Male teenagers had higher incidence rates of risky driving events than female teenagers (AIRR, 1.43; 97.5% CI, 1.13-1.81).

**Table 2.** Unadjusted and Adjusted IRRs of Risky Driving Events Per 1000 Miles Driven Across Study Arms (160 095 Trips by 240 Teenagers)

Risky driving events	Unadjusted IRR (97.5% CI)^a^	*P* value	Adjusted IRR (97.5% CI)^a^^,^^b^	*P* value
Driving feedback only vs control	0.98 (0.73-1.32)	.008^c^	0.98 (0.74-1.31)	.003^c^
Driving feedback plus parent training vs control	0.70 (0.52-0.94)		0.68 (0.51-0.90)	
Driving feedback plus parent training vs driving feedback only	0.71 (0.53-0.95)		0.69 (0.51-0.92)	
Sex (male vs female)	NA	NA	1.43 (1.13-1.81)	.006^c^
Age (17 vs 16 y)	NA	NA	1.12 (0.88-1.41)	.29^c^

Abbreviations: IRR, incidence rate ratio; NA, not applicable.

^a^ IRR was estimated using the trip-based mixed-effects zero-inflated negative binomial regression model’s count component, with miles driven as an offset.

^b^ Adjusting for teenagers’ sex and age.

^c^ Calculated using a χ^2^ test comparing differences across the 3 arms. A significance threshold of *P* < .025 was used, based on a Bonferroni adjustment for 2 prespecified coprimary end points. The corresponding 97.5% CIs were also Bonferroni adjusted.

The proportion of miles driven while speeding was significantly lower in both intervention arms than in the control arm in both unadjusted and adjusted models (Table 3). Compared with the control arm, teenagers in the driving feedback only arm had a 36% (adjusted exponentiated β coefficient, 0.64; 97.5% CI, 0.54-0.79) lower mean proportion of miles driven while speeding, and teenagers in the driving feedback plus parent training arm had a 46% (adjusted exponentiated β coefficient, 0.54; 97.5% CI, 0.47-0.68) lower mean proportion of miles driven while speeding. Male teenagers had a 20% higher proportion of miles driven while speeding than female teenagers (adjusted exponentiated mean β coefficient, 1.20; 97.5% CI, 1.04-1.42). No other statistically significant differences were observed.

**Table 3.** Unadjusted and Adjusted PRs of Miles Driven Involving Speeding, Across Study Arms (160 095 Trips by 240 Teenagers)

Miles driven involving speeding	Unadjusted PR (97.5% CI)^a^	*P* value	Adjusted PR (97.5% CI)^a^^,^^b^	*P* value
Driving feedback only vs control	0.64 (0.53-0.79)	<.001^c^	0.64 (0.54-0.79)	<.001^c^
Driving feedback plus parent training vs control	0.55 (0.46-0.68)		0.54 (0.47-0.68)	
Driving feedback plus parent training vs driving feedback only	0.87 (0.73-1.08)		0.86 (0.73-1.07)	
Sex (male vs female)	NA	NA	1.20 (1.04-1.42)	.008^c^
Age (17 vs 16 y)	NA	NA	1.01 (0.85-1.17)	.89^c^

Abbreviations: NA, not applicable; PR, proportion ratio.

^a^ The exponentiated β coefficient (multiplicative effect on the mean proportion) was estimated using the trip-based mixed-effects zero-inflated β regression model’s proportion component.

^b^ Adjusting for teenagers’ sex and age.

^c^ Calculated using a χ^2^ test comparing differences across the 3 arms. A significance threshold of *P* < .025 was used, based on a Bonferroni adjustment for 2 prespecified coprimary end points. The corresponding 97.5% CIs were also Bonferroni adjusted.

### Specific Event Rate Ratios and Speeding Proportion Ratios

Exploratory analyses showed that teenagers in the driving feedback plus parent training arm had lower rates of hard braking (AIRR, 0.77; 95% CI, 0.58-1.01) and speed greater than 75 miles per hour (AIRR, 0.81; 95% CI, 0.54-1.22) per 1000 miles than those in the control arm, although these differences were not statistically significant (eTable 3 in Supplement 2). Male teenagers had higher rates of hard braking (AIRR, 1.39; 95% CI, 1.11-1.73) and sudden acceleration (AIRR, 2.13; 95% CI, 1.42-3.19) than female teenagers.

Both intervention arms had significantly lower proportions of miles driven at speeds greater than 75 miles per hour than the control arm (eTable 4 in Supplement 2). Teenagers in the driving feedback only arm had a 21% lower mean proportion (adjusted exponentiated β coefficient, 0.79; 95% CI, 0.67-0.98), and those in the driving feedback plus parent training arm had a 22% lower mean proportion (adjusted exponentiated β coefficient, 0.78; 95% CI, 0.65-0.96). Results from the zero-inflation components of the mixed-effects models showed similar patterns and are reported in eTables 5, 6, 7, and 8 in Supplement 2.

## Discussion

In this randomized clinical trial of teenage drivers (aged 16-17 years) with traffic violations, combining driving feedback with parent training significantly reduced risky driving event rates and miles driven while speeding. Driving feedback alone did not reduce risky driving events but did reduce miles driven while speeding, suggesting that feedback may shorten speeding miles without preventing it and that pairing driving feedback with parent training may be necessary to meaningfully reduce risky driving, behaviors associated with increased crash risk and severity.^40,45,46,47,48^ A key innovation of this study was integrating driving feedback with parent training. Parents were coached in motivational interviewing techniques (OARS) to guide nonjudgmental, data-informed conversations that reinforced safe driving.^15,16,17,18^ This approach empowered parents to support teenager behavior change and, consistent with prior research,^9,14,16,19,20^ underscores the importance of continued parental involvement after supervised driving ends.

Our finding that driving feedback alone reduced persistent speeding, but not overall risky driving events, adds nuance to the literature on in-vehicle feedback technology. Although such systems have reduced risky driving among commercial drivers,^49,50^ results among teenagers have been mixed.^17,26,27,28,29,30^ This inconsistency may reflect differences in the target population,^30,31^ study design,^26,27^ type of feedback (eg, visual vs audio alerts),^17^ and feedback recipients (teenagers only vs teenagers and parents).^28,29^ Most prior studies of feedback interventions among teenagers have focused on risky driving and speeding event rates, without examining speeding duration. In contrast, our study assessed both event rates and speeding duration, allowing us to detect reductions in persistent speeding even when event rates did not change. Prior studies show that feedback interventions are most effective when immediate alerts to teenagers are paired with delayed feedback to parents; alerts alone are insufficient.^17,26,27,28,29,30^ Our findings support this and highlight the importance of pairing in-vehicle feedback with active parental involvement.^51,52^

Consistent with prior research showing that approximately 89% of adolescent trips involved no kinematic risky driving events,^39^ we found that 78% of trips in our sample had no risky driving events, and 83% had no kinematic events. Also aligning with earlier evidence,^1,3^ our results reveal notable sex differences in teenage driving behaviors, with male teenagers exhibiting higher incidence rates of overall risky driving, hard braking, and sudden acceleration, as well as greater proportions of miles driven while speeding, than female teenagers. These disparities underscore the need for targeted interventions to reduce crash risk among male teenage drivers. Tailored strategies, such as embedding interventions with juvenile traffic court programs, may help mitigate risky behaviors and enhance safety after traffic citations.

### Limitations

This study has several limitations. The sample, drawn from 6 Ohio counties, was predominantly White, English-speaking, and well educated, limiting generalizability; requiring regular vehicle access likely contributed to more than 80% of participating households with incomes above the state median of $69 680. Engagement with feedback reports, parental use of training materials, and parent-teenager communication were not directly assessed, and driving data may have included trips by nonparticipant drivers. In addition, seatbelt nonuse could not be detected for all vehicles. Despite these limitations, this study advances the literature by targeting teenagers with traffic violations, integrating in-vehicle feedback with parent training, and using objective driving measures to evaluate intervention effectiveness.

## Conclusions

This randomized clinical trial demonstrates that combining in-vehicle feedback with parent training and access to teenage driving reports improves driving behaviors among teenagers with traffic violations, whereas feedback alone did not reduce risky driving events despite reducing persistent speeding. These findings highlight the importance of sustained parental involvement in promoting safe driving after licensure, particularly after a traffic citation. Clinicians can reinforce teenage driving safety by encouraging parents to remain engaged and to use feedback tools that support safe driving. Future research should support adopting juvenile traffic court referral programs that integrate in-vehicle feedback and parent communication training to improve teenage driving safety.

## Notes

Supplement 1.
Trial Protocol and Statistical Analysis Plan

Supplement 2. **eTable 1.** Proportion of Miles Driven With Detected Seatbelt Nonuse (n = 83) **eTable 2.** Self-Reported Distracted Driving Behaviors During the Past Two Weeks **eTable 3.** Incidence Rate Ratios (Unadjusted and Adjusted) of Specific Risky Driving Events per 1,000 Miles Driven Across Study Arms (n = 160,095 Trips by 240 Teens) **eTable 4.** Proportion Ratios (Unadjusted and Adjusted) of Miles Driven Involving Specific Speeding Behaviors Across Study Arms (n = 160,095 Trips by 240 Teens) **eTable 5.** Odds Ratios (Unadjusted and Adjusted) of Risky Driving Events Across Study Arms (n = 160,095 Trips by 240 Teens) **eTable 6.** Odds Ratios (Unadjusted and Adjusted) of Miles Driven Involving Speeding Behaviors Across Study Arms (n = 160,095 Trips by 240 Teens) **eTable 7.** Odds Ratios (Unadjusted and Adjusted) of Specific Risky Driving Events Across Study Arms (n = 160,095 Trips by 240 Teens) **eTable 8.** Odds Ratios (Unadjusted and Adjusted) of Miles Driven Involving Specific Speeding Behaviors Across Study Arms (n = 160,095 Trips by 240 Teens)

Supplement 3.
Data Sharing Statement

## References

[1] Teen drivers. National Center for Injury Prevention and Control; Centers for Disease Control and Prevention. January 29, 2026. Accessed March 12, 2026. https://www.cdc.gov/teen-drivers/about/index.html

[2] Mayhew DR, Simpson HM, Pak A. Changes in collision rates among novice drivers during the first months of driving. Accid Anal Prev. 2003;35(5):683-691. doi:10.1016/S0001-4575(02)00047-7 12850069

[3] Fatality facts 2023: teenagers. Insurance Institute for Highway Safety (IIHS). Accessed March 12, 2026. https://www.iihs.org/topics/fatality-statistics/detail/teenagers

[4] Peek-Asa C, Zhang L, Hamann CJ, O’Neal E, Yang J. Direct medical charges of all parties in teen-involved vehicle crashes by culpability. Inj Prev. 2023;29(4):334-339. doi:10.1136/ip-2022-044841 37147120 PMC10583597

[5] Alderman EM, Johnston BD; Committee on Adolescence; Council on Injury, Violence, and Poison Prevention. The teen driver. Pediatrics. 2018;142(4):e20182163. doi:10.1542/peds.2018-2163 30249622

[6] Zakrajsek JS, Shope JT, Greenspan AI, Wang J, Bingham CR, Simons-Morton BG. Effectiveness of a brief parent-directed teen driver safety intervention (Checkpoints) delivered by driver education instructors. J Adolesc Health. 2013;53(1):27-33. doi:10.1016/j.jadohealth.2012.12.010 23481298 PMC4147835

[7] Mirman JH, Curry AE, Winston FK, . Effect of the teen driving plan on the driving performance of teenagers before licensure: a randomized clinical trial. JAMA Pediatr. 2014;168(8):764-771. doi:10.1001/jamapediatrics.2014.252 24957844

[8] Winston FK, Mirman JH, Curry AE, Pfeiffer MR, Elliott MR, Durbin DR. Engagement with the TeenDrivingPlan and diversity of teens’ supervised practice driving: lessons for internet-based learner driver interventions. Inj Prev. 2015;21(1):4-9. doi:10.1136/injuryprev-2014-041212 24916684

[9] Hafetz J, McDonald CC, Long DL, . Promoting transportation safety in adolescence: the Drivingly randomized controlled trial. BMC Public Health. 2023;23(1):2020. doi:10.1186/s12889-023-16801-6 37848929 PMC10580546

[10] Factor R. The effect of traffic tickets on road traffic crashes. Accid Anal Prev. 2014;64:86-91. doi:10.1016/j.aap.2013.11.010 24342150

[11] Summala H, Rajalin S, Radun I. Risky driving and recorded driving offences: a 24-year follow-up study. Accid Anal Prev. 2014;73:27-33. doi:10.1016/j.aap.2014.08.008 25171522

[12] Ekeh AP, Hamilton SB, D’Souza C, Everrett E, McCarthy MC. Long-term evaluation of a trauma center–based juvenile driving intervention program. J Trauma. 2011;71(1):223-226. doi:10.1097/TA.0b013e31821cc0fd 21818028

[13] Baird J, Nirenberg TD, Longabaugh R, Mello MJ. The effect of group-adapted motivational interviewing on traffic convictions and driving behaviors of court-adjudicated youth. Traffic Inj Prev. 2013;14(6):572-577. doi:10.1080/15389588.2012.734666 23859670

[14] Curry AE, Peek-Asa C, Hamann CJ, Mirman JH. Effectiveness of parent-focused interventions to increase teen driver safety: a critical review. J Adolesc Health. 2015;57(1 suppl):S6-S14. doi:10.1016/j.jadohealth.2015.01.003 26112737 PMC4483193

[15] Ramirez M, Yang J, Young T, . Implementation evaluation of *Steering Teens Safe*: engaging parents to deliver a new parent-based teen driving intervention to their teens. Health Educ Behav. 2013;40(4):426-434. doi:10.1177/1090198112459517 23041706

[16] Hamann C, Schwab-Reese L, O’Neal EE, Butcher B, Yang J, Peek-Asa C. Family communication patterns and teen driving intervention effectiveness. Am J Health Behav. 2019;43(5):963-975. doi:10.5993/AJHB.43.5.8 31439102 PMC7654442

[17] Peek-Asa C, Reyes ML, Hamann CJ, Butcher BD, Cavanaugh JE. A randomized trial to test the impact of parent communication on improving in-vehicle feedback systems. Accid Anal Prev. 2019;131:63-69. doi:10.1016/j.aap.2019.06.006 31233996

[18] Peek-Asa C, Cavanaugh JE, Yang J, Chande V, Young T, Ramirez M. Steering teens safe: a randomized trial of a parent-based intervention to improve safe teen driving. BMC Public Health. 2014;14:777. doi:10.1186/1471-2458-14-777 25082132 PMC4125695

[19] Simons-Morton B, Ouimet MC. Parent involvement in novice teen driving: a review of the literature. Inj Prev. 2006;12(suppl 1):i30-i37. doi:10.1136/ip.2006.011569 16788109 PMC2563441

[20] Simons-Morton B. Parent involvement in novice teen driving: rationale, evidence of effects, and potential for enhancing graduated driver licensing effectiveness. J Safety Res. 2007;38(2):193-202. doi:10.1016/j.jsr.2007.02.007 17478190 PMC1942043

[21] Mattox J. The Impact of a Court-Directed Parental Involvement Program on Parental Monitoring and Young-Driver Behaviors. Dissertation. The University of Memphis; 1999.

[22] Manno M, Maranda L, Rook A, Hirschfeld R, Hirsh M. The reality of teenage driving: the results of a driving educational experience for teens in the juvenile court system. J Trauma Acute Care Surg. 2012;73(4)(suppl 3):S267-S272. doi:10.1097/TA.0b013e31826b00f4 23026966

[23] Nirenberg T, Baird J, Longabaugh R, Mello MJ. Motivational counseling reduces future police charges in court referred youth. Accid Anal Prev. 2013;53:89-99. doi:10.1016/j.aap.2013.01.006 23384442 PMC3594417

[24] Watson A, Kaye SA, Fleiter J, Freeman J. Effectiveness of vehicle impoundment for high-range speeding offences in Victoria, Australia. Accid Anal Prev. 2020;145:105690. doi:10.1016/j.aap.2020.105690 32711215

[25] Winston FK, Puzino K, Romer D. Precision prevention: time to move beyond universal interventions. Inj Prev. 2016;22(2):87-91. 26271260 doi:10.1136/injuryprev-2015-041691 26271260

[26] McGehee DV, Raby M, Carney C, Lee JD, Reyes ML. Extending parental mentoring using an event-triggered video intervention in rural teen drivers. J Safety Res. 2007;38(2):215-227. doi:10.1016/j.jsr.2007.02.009 17478192

[27] Carney C, McGehee DV, Lee JD, Reyes ML, Raby M. Using an event-triggered video intervention system to expand the supervised learning of newly licensed adolescent drivers. Am J Public Health. 2010;100(6):1101-1106. doi:10.2105/AJPH.2009.165829 20395588 PMC2866618

[28] Farmer CM, Kirley BB, McCartt AT. Effects of in-vehicle monitoring on the driving behavior of teenagers. J Safety Res. 2010;41(1):39-45. doi:10.1016/j.jsr.2009.12.002 20226949

[29] Simons-Morton BG, Bingham CR, Ouimet MC, . The effect on teenage risky driving of feedback from a safety monitoring system: a randomized controlled trial. J Adolesc Health. 2013;53(1):21-26. doi:10.1016/j.jadohealth.2012.11.008 23375825 PMC3644526

[30] Farah H, Musicant O, Shimshoni Y, . Can providing feedback on driving behavior and training on parental vigilant care affect male teen drivers and their parents? Accid Anal Prev. 2014;69:62-70. doi:10.1016/j.aap.2013.11.005 24331278

[31] Shimshoni Y, Farah H, Lotan T, . Effects of parental vigilant care and feedback on novice driver risk. J Adolesc. 2015;38:69-80. doi:10.1016/j.adolescence.2014.11.002 25480357

[32] Kaur A, Williams J, Recker R, Rose D, Zhu M, Yang J. Subsequent risky driving behaviors, recidivism and crashes among drivers with a traffic violation: a scoping review. Accid Anal Prev. 2023;192:107234. doi:10.1016/j.aap.2023.107234 37556998 PMC10634619

[33] Rose DM, Sieck CJ, Kaur A, Wheeler KK, Sullivan L, Yang J. Factors influencing participation and engagement in a teen safe driving intervention: a qualitative study. Int J Environ Res Public Health. 2024;21(7):928. doi:10.3390/ijerph21070928 39063504 PMC11276654

[34] Yang J, Peek-Asa C, Zhang Y, . ProjectDRIVE: study protocol for a randomized controlled trial to improve driving practices of high-risk teen drivers with a traffic violation. Inj Epidemiol. 2024;11(1):12. doi:10.1186/s40621-024-00494-5 38553746 PMC10979602

[35] Hopewell S, Chan AW, Collins GS, . CONSORT 2025 statement: updated guideline for reporting randomized trials. JAMA. 2025;333(22):1998-2005. doi:10.1001/jama.2025.4347 40228499

[36] World Medical Association. World Medical Association Declaration of Helsinki: ethical principles for medical research involving human subjects. JAMA. 2013;310(20):2191-2194. doi:10.1001/jama.2013.281053 24141714

[37] Juvenile Traffic Court. Franklin County, Ohio, Court of Common Pleas. Accessed March 12, 2026. https://drj.fccourts.org/Court-Services/Juvenile-Court/Juvenile-Traffic-Court

[38] Fleet tracking software. Azūga Inc. Accessed March 12, 2026. https://www.azuga.com/fleet-tracking

[39] McDonald CC, Rix K, Ebert JP, . Handheld cellphone use and risky driving in adolescents. JAMA Netw Open. 2024;7(10):e2439328. doi:10.1001/jamanetworkopen.2024.39328 39418022 PMC11581603

[40] Simons-Morton BG, Zhang Z, Jackson JC, Albert PS. Do elevated gravitational-force events while driving predict crashes and near crashes? Am J Epidemiol. 2012;175(10):1075-1079. doi:10.1093/aje/kwr440 22271924 PMC3353134

[41] Resnicow K, Jackson A, Wang T, . A motivational interviewing intervention to increase fruit and vegetable intake through Black churches: results of the Eat for Life trial. Am J Public Health. 2001;91(10):1686-1693. doi:10.2105/AJPH.91.10.1686 11574336 PMC1446855

[42] Berg-Smith SM, Stevens VJ, Brown KM, ; The Dietary Intervention Study in Children (DISC) Research Group. A brief motivational intervention to improve dietary adherence in adolescents. Health Educ Res. 1999;14(3):399-410. doi:10.1093/her/14.3.399 10539230

[43] Rollnick S. Motivational Interviewing: Preparing People for Change. Guilford Press; 2002.

[44] Feng CX. A comparison of zero-inflated and hurdle models for modeling zero-inflated count data. J Stat Distrib Appl. 2021;8(1):8. doi:10.1186/s40488-021-00121-4 34760432 PMC8570364

[45] Ehsani JP, Gershon P, Grant BJB, . Learner driver experience and teenagers’ crash risk during the first year of independent driving. JAMA Pediatr. 2020;174(6):573-580. doi:10.1001/jamapediatrics.2020.0208 32250391 PMC7136860

[46] Lin C, Wu D, Liu H, Xia X, Bhattarai N. Factor identification and prediction for teen driver crash severity using machine learning: a case study. Appl Sci (Basel). 2020;10(5):1675. doi:10.3390/app10051675

[47] Duddu VR, Kukkapalli VM, Pulugurtha SS. Crash risk factors associated with injury severity of teen drivers. IATSS Res. 2019;43(1):37-43. doi:10.1016/j.iatssr.2018.08.003

[48] Simons-Morton BG, Ouimet MC, Zhang Z, . Crash and risky driving involvement among novice adolescent drivers and their parents. Am J Public Health. 2011;101(12):2362-2367. doi:10.2105/AJPH.2011.300248 22021319 PMC3222425

[49] Ghamari A, Rezaei N, Malekpour MR, . The effect of non-punitive peer comparison and performance feedback on drivers’ behavior using the telematics: the first randomized trial in Iran. J Safety Res. 2022;82:430-437. doi:10.1016/j.jsr.2022.07.010 36031273

[50] Horrey WJ, Lesch MF, Dainoff MJ, Robertson MM, Noy YI. On-board safety monitoring systems for driving: review, knowledge gaps, and framework. J Safety Res. 2012;43(1):49-58. doi:10.1016/j.jsr.2011.11.004 22385740

[51] Weiss E, Fisher Thiel M, Sultana N, Hannan C, Seacrist T. Advanced driver assistance systems for teen drivers: teen and parent impressions, perceived need, and intervention preferences. Traffic Inj Prev. 2018;19(sup1):S120-S124. doi:10.1080/15389588.2017.1401220 29584476

[52] Bishop HJ, O’Donald M, O’Malley L, . A novel technological approach to preventing distracted driving. J Safety Res. 2025;93:24-30. doi:10.1016/j.jsr.2025.02.003 40483059 PMC13137913

