# Supplementary material for: In-Vehicle Feedback With or Without Parent Communication Training and Teenage Driving Behaviors: A Randomized Clinical Trial
Source: JAMA Netw Open. 2026 Apr 24;9(4):e268631. doi: 10.1001/jamanetworkopen.2026.8631 (PMC13109799; doi:10.1001/jamanetworkopen.2026.8631)
Supplement: Supplement 1. — Trial Protocol and Statistical Analysis Plan [file jamanetwopen-e268631-s001.pdf]

Supplement 1 eProtocol

PROTOCOL TITLE:
Intervention to Improve Driving Practices Among High-Risk Teen Drivers

PRINCIPAL INVESTIGATOR:
Name: Jingzhen (Ginger) Yang
Department/Center: Center for Injury Research and Policy
Telephone Number: 614-355-5852
Email Address: ginger.yang@nationwidechildrens.org

1.0 Study Summary

**Study Title**	Intervention to Improve Driving Practices Among High-Risk Teen Drivers
**Study Design**	Clinical Trial
**Primary Objective**	Determine the effects of the intervention on teens' risky driving events and unsafe driving behaviors.
**Secondary Objective(s)**	Determine the effects of the intervention on frequency and quality of parent-teen communications about safe driving practices, and traffic violation recidivism.
**Research Intervention(s)/ Investigational Agent(s)**	Driving feedback and parental communication training.
**IND/IDE #**	
**Study Population**	Youth aged 16-17 who committed a moving-related traffic violation and their parent/legal guardian ('parent') most involved with their driving.
**Sample Size**	240
**Study Duration for individual participants**	6 months
**Study Specific Abbreviations/ Definitions**	

2.0 Objectives

2.1 Describe the purpose, specific aims, or objectives.
Aim 1: Determine the effects of the intervention on teens' risky driving events and unsafe driving behaviors.
Aim 2: Determine the effects of the intervention on frequency and quality of parent-teen communications about safe driving practices.
Aim 3: Determine the effects of the intervention on teen traffic violation recidivism.

2.2 State the hypotheses to be tested.
Aim 1: Hypotheses: The two intervention groups will demonstrate significant reductions in risky driving events (hard braking, sudden acceleration, speeding) and unsafe driving behaviors (speeding, no seatbelt use, distracted driving) per 1,000 miles driven when compared to the control group; The Driving-Feedback plus Parent Communication Training group will have fewer risky driving events and unsafe driving behaviors than the Driving Feedback Only group.
Aim 2: Hypothesis: The Driving-Feedback plus Parent Communication Training group will engage in more frequent and higher quality parent-teen communications on safe-driving topics than the other two groups.
Aim 3: Hypothesis: The two intervention groups will demonstrate significant reductions in recidivism (subsequent traffic violations) when compared to the Control group; The Driving-Feedback plus Parent Communication Training group will have lower rates of recidivism than the Driving Feedback Only group.

3.0 Background

3.1 Describe the relevant prior experience and gaps in current knowledge.
Motor vehicle crashes are the leading cause of injury and death among teenagers in the US, and teen drivers have higher crash rates than drivers in any other age group.(1-6) Drivers who have committed a traffic violation are a particularly high-risk group for crashes, and crash-related injuries and deaths.(7-15) A growing

number of technological advancements, ranging from in-vehicle devices to smartphone applications, are available to monitor and provide real-time and cumulative driving feedback to teens in order to reduce risky driving events and unsafe behaviors.(16-18) Evidence also suggests that parents play a vital role in their teens' safe driving behaviors by reinforcing safe driving habits and setting rules that limit driving in risky conditions. However, many parent-based interventions that utilize technology are limited to providing only feedback on driving, without actively instructing parents how to use the feedback. (17,19-21) Furthermore, very few teen driving interventions are designed specifically for parents of juvenile traffic offenders.

References:

1. Centers for Disease Control and Prevention. Data and Statistics (WISQARS). www.cdc.gov/injury/wisqars. Accessed February 1, 2016.
2. Insurance Institute for Highway Safety (IIHS). Fatality facts: teenagers 2013. Arlington, VA: http://www.iihs.org/iihs/topics/t/teenagers/fatalityfacts/teenagers. Accessed February 1, 2016.
3. Williams AF. Teenage drivers: patterns of risk. J Safety Res. 2003;34(1):5-15. PMID: 12535901.
4. Mayhew DR, Simpson HM, Pak A. Changes in collision rates among novice drivers during the first months of driving. Accid Anal Prev. 2003;35(5):683-691. PMID:12850069.
5. McCartt AT, Shabanova VI, and Leaf WA. Driving experiences, crashes, and teenage beginning drivers. Accid Anal Prev. 2003;35(3):311-320. PMID: 12643948.
6. Jonah BA, and Dawson NE. Youth and risk: age differences in risky driving, risk perception, and risk utility. Alcohol, Drugs and Driving. 1987;3(3):13-29.
7. Summala H, Rajalin S and Radun I. Risky driving and recorded driving offences: a 24-year follow-up study. Accident; analysis and prevention. 2014;73:27-33.
8. Alver Y, Demirel MC, and Mutlu MM. Interactions between socio-demographic characteristics: traffic rule violations and traffic crash history for young drivers. Accident analysis and prevention. 2014;72:95-104.
9. Ayuso M, Guillén M, and Alcaniz M. The impact of traffic violations on the estimated cost of traffic accidents with victims. Accident analysis and prevention. 2010;42(2):709–717.
10. Factor R. The effect of traffic tickets on road traffic crashes. Accident analysis and prevention. 2014;64:86-91.
11. Gebers MA, and Peck RC. Using traffic conviction correlates to identify high accident-risk drivers. Accident analysis and prevention. 2003;35:903–912.
12. Goldenbed C, Reurings M, Van Norden Y, and Stipdonk H. Crash involvement of motor vehicles in relationship to the number and severity of traffic offenses. An exploratory analysis of Dutch traffic offenses and crash data. Traffic injury prevention. 2013;14(6):584-591.
13. Chen W, Cooper P, and Pinili M. Driver accident risk in relation to the penalty point system in British Columbia. J Safety Res. 1995;26:9–18.
14. Cooper PJ. The relationship between speeding behaviour (as measured by violation convictions) and crash involvement. J Safety Res. 1997;28:83–95.
15. Rajalin S. The connection between risky driving and involvement in fatal accidents. Accident analysis and prevention. 1994;26(5):555–562.
16. Carney C, McGehee DV, Lee JD, Reyes ML and Raby M. Using an event-triggered video intervention system to expand the supervised learning of newly licensed adolescent drivers. American journal of public health. 2010;100:1101-6.
17. Farmer CM, Kirley BB and McCartt AT. Effects of in-vehicle monitoring on the driving behavior of teenagers. Journal of safety research. 2010;41:39-45.
18. McGehee DV, Raby M, Carney C, Lee JD and Reyes ML. Extending parental mentoring using an event-triggered video intervention in rural teen drivers. Journal of safety research. 2007;38:215-27.
19. McCartt AT, Farmer CM and Jenness JW. Perceptions and experiences of participants in a study of in-vehicle monitoring of teenage drivers. Traffic injury prevention. 2010;11:361-70.
20. Curry AE, Peek-Asa C, Hamann CJ and Mirman JH. Effectiveness of Parent-Focused Interventions to Increase Teen Driver Safety: A Critical Review. The Journal of adolescent health: official publication of the Society for Adolescent Medicine. 2015;57:S6-14.
21. Farah H, Musicant O, Shimshoni Y, Toledo T, Grimberg E, Omer H and Lotan T. Can providing feedback on driving behavior and training on parental vigilant care affect male teen drivers and their parents? Accident; analysis and prevention. 2014;69:62-70.

3.2 Describe any relevant preliminary data.

We tested the feasibility of using in-vehicle driving feedback technology paired with parent communication training in a pilot in September 2017 at the Franklin County Juvenile Traffic Court. Teens and their parents were approached by a member of our research team following the teen's court hearing and invited to participate in the study. Interested parent-teen dyads were then screened for eligibility and then enrolled, if eligible. We enrolled 10 dyads (77% participation rate, 60% male, 90% first offense) within one week. Once

the in-vehicle device and app were successfully installed/downloaded, we collected three weeks of driving data for each teen. Additionally, each parent was provided an individualized, virtual communication training (one week after enrollment) and a booster session (four weeks after enrollment) with a traffic safety communication specialist experienced in delivering the training. We instructed parents to review their teen's cumulative driving data and an online version of the parent-teen safe driving communication guide. Parents were also provided with a voice recorder to record and submit a 3-minute parent-teen conversation about safe driving following the initial parent communication training session. This recorded parent-teen conversation was used by the communication specialist to tailor the respective booster sessions and to assess intervention fidelity.

3.3 Provide the scientific or scholarly background for, rationale for, and significance of the research based on the existing literature and how will it add to existing knowledge.

Our study will be the first to simultaneously evaluate the effectiveness of novel driving feedback technology augmented with parent communication training to improve safe driving practices among teens with traffic violations. Building on health-related behavioral change and health communication theories, this intervention trial will fill a critical gap in universal approaches by focusing on a high-risk population thus informing development of NIH-defined indicated level interventions. This study will have a significant impact on scientific knowledge by determining whether teen driving practices can be improved by driving feed-back technology and how parents can be assisted by the technology and training in reinforcing safe driving habits of teens following a traffic violation. This study will also provide evidence to inform traffic court judges' decisions regarding the type of driving safety program to which they refer teens. If our hypothesis is confirmed, we anticipate wide dissemination of our designed intervention throughout juvenile traffic offense systems, as well as the translation of this intervention to other high-risk teen driver populations and settings

4.0 Study Endpoints

4.1 Describe the primary and secondary study endpoints.

Determine the effects of the intervention on teens' risky driving events and unsafe driving behaviors.

Determine the effects of the intervention on frequency and quality of parent-teen communications about safe driving practices and traffic violation recidivism.

5.0 Study Intervention/Investigational Agent

5.1 Description: Describe the study intervention and/or investigational agent (e.g., drug, device) that is being evaluated.

In-vehicle Driving Feedback Technology: The Azūga™ in-vehicle driving feedback technology, which consists of a pager-sized device plugged into the vehicle's on-board diagnostic port (installed in the teen's car) and a smartphone app (downloaded on the teen's smartphone), will be installed/downloaded. Four types of feedback will be provided to intervention teens: 1) Direct audio feedback from the installed device; 2) Push notification on the phone screen when a trip ends; 3) Detailed cumulative driving data; and 4) A customized biweekly driving summary report.

Parent Training: An individualized virtual training in communication strategies about driving safety along with a booster session will be delivered by a traffic safety communication specialist to parents in the Driving Feedback plus Parent Communication Training group. Parents in this group will also be provided with access to their teen's driving data as well as an online parent-teen safe driving communication guide.

5.2 Device Handling: If the research involves drugs or device, describe your plans to store, handle, and administer those drugs or devices so that they will be used only on subjects and be used only by authorized investigators.

N/A

5.3 If the drug is investigational (has an IND) or the device has an IDE or a claim of abbreviated IDE (non-significant risk device), include the following information:

N/A

6.0 Procedures Involved*

6.1 Describe and explain the study design.

This study is a randomized controlled trial (RCT) with one control and two intervention groups. Participants will include 240 dyads (80 per group), comprised of one teen driver (ages 16 or 17) who has committed a

moving violation (e.g., speeding, failure to obey traffic signal) during the recruitment period and the parent/legal guardian (‘parent’) who is most involved with the teen’s driving. According to Ohio law, when a teen driver under 18 years is cited by law enforcement for a traffic violation, the teen must appear in Juvenile Traffic Court within 2 weeks of the citation accompanied by a parent to confess or deny committing the traffic violation, providing our team with a good opportunity to recruit and enroll participants. After completing informed consent/assent and the baseline assessment, enrolled dyads will be randomly assigned into 1 of 3 groups using a stratified block randomization.

6.2 Provide a description of all research procedures being performed and when they are performed, including procedures being performed to monitor subjects for safety or minimize risks.

In-person recruitment and enrollment: If interested in participating in the study, participants can notify the Nationwide Children’s Hospital (NCH) on-site research team after their court hearing. The NCH research team member will then talk more about the study, answer any questions, screen for eligibility, obtain written consent/assent, and schedule an in-person meeting at a place that is convenient for participants. Participants will be ensured that their refusal to participate in this study will not affect their driving privileges in any way, and that the judge and court official will be blinded to who participates in our research study. Subjects will then be randomized into one of the three study groups (described in detail later).

During the initial scheduled meeting, the NCH research team member will install the in-vehicle device in the teen’s car and download the smartphone app on the teen’s smartphone. The in-vehicle device will stay in the car for a period of six months. Teens in the Control Group will have the in-vehicle device installed in their car, but all feedback features will be disabled. If they are randomized into the Feedback Only Group, researchers will provide instructions on how teens can review their driving data. If in the Device Feedback plus Parent Communication Training Group, the NCH research team member will help schedule a video call with a specialist at the University of Iowa within 2 weeks of enrollment for parents to receive communication training on how to motivate their teen to adopt safe driving habits. This video call will be up to 45 minutes long and will take place 2 weeks after enrollment. The research team will also help schedule a booster session 2 months of the initial training session. Additionally, the member of our research team will show the parent how to access their teen’s cumulative driving data. The teens of the Feedback Only Group will also receive a biweekly cumulative driving report. Teens and parents of the Device Feedback Plus Parent Communication Training Group will also receive a biweekly cumulative driving report as well as parental progress completing the online safe driving communication guide. Furthermore, we will obtain information about the teen’s car year, make, and model for all participants regardless of group assignment. This initial meeting should take approximately 30 minutes.

The research team will reach out to participants at 3 months post-enrollment to have them fill out an online survey and terminate the active intervention for the intervention groups (Groups 2 & 3). At the end of 6 months, the NCH research team member will meet with participants in-person to end enrollment, and to collect the in-vehicle device. Additionally, they will administer a final survey to participants. After completion of this survey, participation in this study will be complete.

Virtual recruitment and enrollment: Teen drivers that receive a citation will still come to the courthouse for their hearing. However, teens and parents that are interested in participating will be able to scan a QR code from a study flyer that is posted within the waiting room of the courthouse or receive the flyer with their final paperwork from the clerks. If the teen and parent are interested, they will scan the QR code which will direct them to our REDCap. On the REDCap they will complete the eligibility form. If they are eligible, it will alert the NCH research team. The NCH research team will then reach out to the teen and parent to confirm interest in the study and schedule them for the enrollment meeting. The NCH research team will send the consent form to the teen and parent at this time for them to review prior to the meeting.

During the enrollment meeting, the NCH research team will video conference with the teen and parent. The researcher will go through the consent form and answer any questions the teen or parent may have. Once the questions have been answered the teen and parent will be instructed to virtually sign the consent form. Once they have completed the consent and assent form, the researcher will then send other study documents to the teen and parent through REDCap to complete. Once they have completed those forms the researcher will describe the specific group activities the teen and parent will need to complete based on which group they are in for the study. At the end of the call, the researcher will schedule another call with the teen and parent for the installation of the study devices. Once the call has ended the researcher will send any documents the teen or parent requested (i.e., consent form) and the group activities sheet. The researcher will also ship the study devices within 24 hours of the initial call to the teen and parent. This call should take about 30 to 45 minutes.

This study will use Twilio, a third-party platform that is integrated with REDCap, to:
Send SMS messages with survey links to a REDCap form:
Bi-weekly REDCap surveys will be sent via text. The text message will not include PHI or PII. The text message will provide a link to the secure REDCap platform. The participant will click on the link and be directed to a secure REDCap platform to complete the weekly survey.

Send informational texts and notifications:
Participants will be sent informational texts to remind them to complete various aspects of the study. For example, participants may receive a text message two days before the scheduled survey date to remind them about the upcoming survey or contact the research team. These text messages will not include PHI or PII.
Prior to the second meeting, the researcher will also make sure to send installation instructions if the teen and parent are interested in installing the devices before the next call. Once the teen and parent have received the study devices, there will be another video conference. During this meeting, the researcher will check to make sure that the devices and apps have been correctly installed and downloaded for the teen and parent. If the devices have not been installed, the researcher will help walk the teen and parent through the process. Once the devices have been installed the researcher will remind the teen and parent of any forms they did not complete. If they still have not completed them by the following week an email will be sent to the teen and parent as a reminder. This call should take about 30 minutes to an hour.

6.3 Describe:

6.3.1 Procedures performed to lessen the probability or magnitude of risks.

All data collected will only be accessible to members of our research team. Data collected will be securely transferred to a protected research server at NCH and will be password-protected.

A Data and Safety Monitoring Committee (DSMC), comprised of an expert in Clinical and Translational Research with expertise in traffic injury and safety, a clinical psychologist who specializes in coping and adjustment issues for adolescents and their families, an injury researcher who specializes in technological applications and teen driving safety, and a state official who leads injury prevention efforts in the state of Ohio, has been established for the proposed study. The DSMC will collaborate with our research team throughout the duration of the study.

The secure Azūga™ Amazon Web server and the secure NCH server will be utilized to protect all study data, and in particular data collected from the in-vehicle device (e.g., GPS data). Several precautions will be taken to protect participant confidentiality and privacy. These include:

1. Anonymizing participants (only using Azūga™ participant aliases, rather than their real names),
2. Isolating data (storing our data on a physically isolated drive separate from all other Azūga™ clients' data),
3. Controlling access (only our research team will be able to access the data),
4. Monitoring data remotely (designated research staff can monitor participants' data remotely at any time),
5. Keeping individually identifiable private information in a separate file that is only accessible to the PI and authorized researchers,
6. Representing participants by computer-assigned case numbers, instead of names, images, and/or specific identifiers,
7. Maintaining all data on password-protected computers and keeping hard copies of the data in a locked file cabinet only accessible by our research team.

Additionally, we will use advanced computer informatics to ensure data is secure (e.g., epsilon-differential privacy), which provides mathematically rigorous guarantees regarding the presence or absence of a particular user's entry in the database, and fully homomorphic encryption, which enables computation of encrypted data without disclosure to ensure the confidentiality of processed data. NCH also has an established committee, the Privacy and Security Advisory Committee (pSAC), which is dedicated to ensuring that study information, particularly information pertaining to study participants, is securely maintained and follows applicable laws and regulations.

6.3.2 The source records that will be used to collect data about subjects. (Attach all surveys, scripts, and data collection forms.)

See Attached

6.4 What data will be collected during the study and how that data will be obtained.

1. Risky driving events (hard braking, sudden acceleration, speeding): will be collected among teens in all three groups using the Azūga™ in-vehicle device. The number and type of driving event, including hard braking (≤ -

0.45 g-force) sudden acceleration (> 0.35 g-force), and speeding (>10 miles over the posted speed limit), and speed >75 mph will be automatically coded and counted in the system. The rates will be computed by dividing events by miles driven then multiplying by 1,000.

2. Unsafe driving behaviors: Using the Azūga™ in-vehicle device and smartphone app, we will collect data on teen's unsafe driving behaviors (e.g., speeding, no seatbelt use). The duration (e.g., miles driven) and type of driving behaviors, including speeding (>10 miles over the posted speed limit), will be automatically coded and counted in the system. The proportions will be calculated by dividing by miles driven then multiplying by 1,000 (e.g., proportion of 1,000 miles in which speeding). In addition, surveys will collect self-reported distracted driving (e.g., calls made and received, texts sent and viewed while driving, searches for a webpage/app) and seatbelt use (since not all vehicle models are available via Azūga™ in-vehicle device).
3. Parent-teen communication: Frequency of parent-teen communication: Dyads will be asked to rate the frequency of parent-teen conversations on each of the 24 common driving skills/safety principles, discussed in the past month (0=*never* to 3=*often*) and level of success (1=*poor* to 10=*excellent*). Frequency of parent-teen communication scores will range from 0 to 72, with higher scores indicating more frequent communication. Quality of parent-teen communication scores will be calculated by averaging ratings for all skills/principles addressed, and then weighting them based on the maximum score possible, and then recording scores as a percentage (possible range= 1% - 100%). Additionally, quality of parent-teen communication will be assessed using voice recorded dyad conversations (one conversation submitted per survey). Conversations will be coded by two trained coders with established inter-rater reliability who will identify if parents 1) use active listening; 2) use "OARS"; 3) solicit input about the teen's perspective, 4) focus on objective behaviors, and/or 5) express emotional responses, with each item scored individually (0=*never* to 3=*often*). A summary score for each conversation will be calculated.
4. Recidivism will be measured among teens in all three groups by linking traffic citations and court disposition data with the participating teen's driver's license number. Recidivism during the 12 months following study completion, including date and type of violation, and days from index violation to subsequent violation will be analyzed. The time of recidivism may not be observed when the 12-month study participation is completed and thus, it will be considered as censored at month 12 (end of study participation).
5. Engagement with device feedback will be measured among teens in Group 2 (Driving Feedback Only) & Group 3 (Driving Feedback plus Parent Training) and parents in Group 3 via online tracking of the participant's web interface using Google Analytics. The number of times each of the driving summaries is accessed (links clicked), and the amount of time spent at each link will be recorded.
6. Engagement with the parent communication training will be measured among parents in Group 3 (Driving Feedback plus Parent Communication Training) using a self-report questionnaire and via online tracking. Self-report: Following the individualized training, and at 3- and 6-months follow-ups, parents will report: (i) how frequently they have used communication strategies in their parent-teen discussions on safe driving during the past month (0=*never* to 3=*often*), (ii) how helpful these strategies and techniques have been (1=*not helpful at all* to 10=*extremely helpful*), and (iii) their perceived level of mastery of the strategies and techniques (1=*poor* to 10=*excellent*). Summary scores will be calculated. Online tracking: Using Google Analytics, parents' interactions with the online parent-teen safe driving communication guide will be tracked, including the number of logins, visited sub-links, and time spent at each link.

7.0 Data and Specimen Banking*

7.1 If data or specimens will be banked for future use, describe where the specimens will be stored, how long they will be stored, how the specimens will be accessed, and who will have access to the specimens.
Data will be stored on a secure password protected computer for 10 years following the completion of the study. Data will only be accessible by research team members on secure password protected computers.

7.2 List the data to be stored or associated with each specimen.
Identifiable data (Names and contact information) will be kept to allow the research team to contact these participants for future studies our research team conducts that they may be eligible for. All de-identified data associated with the study will be kept for future research purposes.

7.3 Describe the procedures to release data or specimens, including: the process to request a release, approvals required for release, who can obtain data or specimens, and the data to be provided with specimens.
Identifiable data will only be released to the research team at NCH or other institution approved to receive PII or PHI as part of the study. A written request for de-identified data must be placed through the PI and/or the required NCH channels. To receive the de-identified data from this study, the requestor must have an IRB that includes the use of the data or the study must be considered non-human research (e.g., analyzing existing data). De-identified data can be obtained by anyone with an approved request.

8.0 Sharing of Results with Subjects*

8.1 Describe whether results (study results or individual subject results, such as results of investigational diagnostic tests, genetic tests, or incidental findings) will be shared with subjects or others (e.g., the subject's primary care physicians) and if so, describe how the results will be shared.

If interested, the final study results will be shared with participants once they are available. Participants will provide an email or mailing address where we can send these results.

9.0 Study Timelines*

9.1 Describe:

9.1.1 The duration of an individual subject's participation in the study.

Participants will be enrolled in the study for a six-month period with 3 months of active intervention.

9.1.2 The duration anticipated to enroll all study subjects.

We anticipate it will take 35 months (study months 7 to 42) to recruit and enroll all participants.

9.1.3 The estimated date for the investigators to complete this study (complete primary analyses)

We anticipate the study to take approximately five years to complete.

10.0 Inclusion and Exclusion Criteria*

10.1 Describe how individuals will be screened for eligibility.

In-person: Individuals will be screened for eligibility by a member of our research team on recruitment days at various Juvenile Traffic Courts in Ohio.

Virtual: Individuals who are interested can scan a QR code from a study flyer that will be placed in the Juvenile Traffic Courts. The QR code will lead the teen and parent to an eligibility form that is on REDCap.

10.2 Describe the criteria that define who will be included or excluded in your final study sample.

The study population will include 240 parent-teen dyads (80 per group), each of which includes a teen (16 or 17 years) with intermediate license who committed a moving violation during the recruitment period (study months 7 to 42) and the parent who is most involved with the teen's driving. Participants will be selected without regard to sex, race/ethnicity, or socioeconomic status.

Inclusion criteria

- Age 16-17 years at time of violation/traffic warning;
- Convicted of a moving violation or received a traffic warning;
- Possess a valid intermediate driver's license issued by the state of Ohio, with proof of car insurance;
- Access to a vehicle with an On-board Diagnostics II system port (i.e., cars made after 1996) in which he/she is the primary driver;
- Smartphone with Bluetooth capabilities;
- At least one legal guardian.

Exclusion criteria

- Unable to drive due to injury, license suspension, or car damage;
- Vehicle already has an in-vehicle driving feedback system installed;
- Extremely low average weekly drive time (e.g. <1 hour per week);
- Currently enrolled in another driving-related study;
- Ward of the State;
- Non-English speaking parent.

10.3 Indicate specifically whether you will include or exclude each of the following special populations: (You may not include members of the above populations as subjects in your research unless you indicate this in your inclusion criteria.)

10.3.1 Adults unable to consent (exclude)
10.3.2 Individuals who are not yet adults (infants, children, teenagers) (include)
10.3.3 Pregnant women (exclude)
10.3.4 Prisoners (exclude)

11.0 Vulnerable Populations*

11.1 If the research involves individuals who are vulnerable to coercion or undue influence, describe additional safeguards included to protect their rights and welfare.

Participating in the study will not affect a participant's court hearing. Clinical services will not be provided as part of this study. To ensure individuals are not coerced to participate in the study by the court, all meetings between participants and research staff, including the initial meeting at the courthouse, will occur only after the teens' court hearing and after they have received the judge's final decision. No court officials will be involved in the recruitment process or implementation of the study protocols and intervention. Additionally, the judge will be blinded from the identity of any teens and parents who choose to participate in the study.

12.0 Local Number of Subjects

12.1 Indicate the total number of subjects to be accrued locally.

240 parent-teen dyads

13.0 Recruitment Methods

13.1 Describe when, where, and how potential subjects will be recruited.

In-person: We will house two research staff members at the courthouse each week (varying by day of week) from 8:30 a.m. to 11:00 a.m., while the juvenile traffic court is in session. On days of recruitment, the teens and their parents will be approached by our on-site research staff members following their scheduled court hearing. The research team member will clearly indicate to the teen and their parent that they are not part of the judicial system. Staff will distribute the study information sheet/flyer to the teen and parent, emphasizing that participation in this study will not impact the court ruling. Interested dyads will then meet our on-site research staff to learn study details, ask questions, be screened for eligibility, and sign consent/assent documents either on-site in a private room or later at a time that is convenient to them. Only dyads with both parent and teen consent/assent will be enrolled. Enrolled participating dyads will then complete a baseline assessment before being randomly assigned into one of three study groups. The judge, who will not be engaged in study recruitment, will be blinded from the identity of dyads who choose to participate in the study and no other court staff will be involved with recruitment for this study. Refusal to participate in this study will have no penalty or loss of benefits to which teen drivers are otherwise entitled and will not affect the outcome of their court hearing in any way.

Virtual: We will have study flyers posted around the waiting room at the Juvenile Traffic Courts. We will share the testimonial video with the courts and other community/school events. Teens and parents who are interested will have the ability to scan the QR code on the flyer which will lead them to our eligibility screening form on REDCap. If the teens and parents are eligible, they will be contacted by our research team to schedule a meeting to virtually sign the consent/assent form and be enrolled in the study. If we contact the potential participant 3 or more times then we will assume they are not interested. We will send them a final email included in the script document. Only dyads with both teen and parent consent/assent will be enrolled. The judge, who will not be engaged in study recruitment, will be blinded from the identity of the dyads who choose to participate in the study. Refusal to participate in this study will have no penalty or loss of benefits to which teen drivers are otherwise entitled and will not affect the outcome of their court hearing in any way.

13.2 Describe the source of subjects.

Our subjects will be drawn from various Juvenile Traffic Courts in Ohio. We will recruit teen drivers aged 16-17 who present to court for their mandated court hearing following a traffic violation.

13.3 Describe the methods that will be used to identify potential subjects.

In-person: Interested dyads will meet our on-site research staff to learn study details, ask questions, and will be screened for eligibility. Researchers will also recruit interested dyads at community/school events, such as Carteens.

Virtual: Interested dyads who are interested will have the ability to scan a QR code that will lead them to our eligibility screening form through REDCap. We will share the testimonial video with courts, collaborators, and other community/school events (e.g., Carteens). Participants will be recruited virtually through multiple online avenues including social media, research databases (e.g., Studysearch, ResearchMatch), and other relevant listservs (e.g. Peachjar).

13.4 Describe materials that will be used to recruit subjects. (Attach copies of these documents with the application).

In-person: Information sheets will be used to describe the study to potential participants.

Virtual: Study flyers designed by NCH will be used to describe the study to potential participants. These flyers will be posted in various community locations such as recreational center, grocery stores, and schools. Approval from the location will be obtained prior to posting the flyer. Testimonial video designed by NCH will be used to share the experiences of parents and teens that completed the study with potential participants.

13.5 Describe the amount and timing of any payments to subjects.

The participating dyad, regardless of group assignment, will receive compensation up to $240 for teen ($30 per month x 6, plus $20 per survey x 3), and $180 for parent ($20 per month x 6, plus $20 per survey x 3). Parents in who receive the communication training will receive an additional $20 for completing a survey after the individualized communication training.

14.0 Withdrawal of Subjects*

14.1 Describe anticipated circumstances under which subjects will be withdrawn from the research without their consent.

There are no anticipated circumstances under which subjects will be withdrawn from the research without their consent.

14.2 Describe any procedures for orderly termination.

There are no procedures for orderly termination.

14.3 Describe procedures that will be followed when subjects withdraw from the research, including partial withdrawal from procedures with continued data collection.

There are no clinical procedures that need to occur if subjects withdraw from the research. If a subject chooses to withdraw from the study, a member of our research team will arrange a time with the subjects to collect the study devices.

15.0 Risks to Subjects*

15.1 List the reasonably foreseeable risks, discomforts, hazards, or inconveniences to the subjects related the subjects' participation in the research. Include as may be useful for the IRB's consideration, a description of the probability, magnitude, duration, and reversibility of the risks. Consider physical, psychological, social, legal, and economic risks.

The potential risks posed to the study participants will be minimal. We acknowledge that there is the potential for emotional stress for the teen participant during the study. However, the stress related to the study is no more than that of day-to-day life. Participants will not be required to answer any questions that they feel uncomfortable answering and will be ensured that their participation in this study is completely voluntary and they are permitted to leave the study at any time. Participants will also be ensured that refusal to participate in this study will have no penalty or loss of benefits to which teen drivers are otherwise entitled. The notifications provided by the study devices (direct feedback beeps and push notifications on app) will be different from what the participants may be accustomed to, however the risk posed by these in-vehicle devices while the subject is driving will be minimal. All information provided by the participant will be kept confidential, and will not be disclosed to family, friends, law enforcement, or the juvenile traffic court.

16.0 Potential Benefits to Subjects*

16.1 Describe the potential benefits that individual subjects may experience from taking part in the research. Include as may be useful for the IRB's consideration, the probability, magnitude, and duration of the potential benefits.

The benefit(s) of the study include teens driving more safely after receiving real-time driving feedback and seeing their driving data and parents learning communication strategies specific to teen driving safety to help motivate their teens to adopt safe driving practices.

17.0 Data Management* and Confidentiality

17.1 Describe the data analysis plan, including any statistical procedures or power analysis.

Our sample size was determined for powering **Aim 1,** based on the primary outcome, the rate of risky driving events using data from our published work, pilot study data, and design parameters. The risky driving events are count data, which will be modeled by Quasi-Poisson distribution with an over-dispersion factor of 25 (based on our pilot study, $\frac{Var}{Mean} = \frac{32.7^2}{43} = 24.9$) for the ordinary Poisson variability. We assume the three groups have the same over-dispersion factor for the Quasi-Poisson distributed data. With these design parameters and 3 groups of an equal sample size of 74, this study will have 0.80 or higher power to detect that Group 2 will have fewer risky driving events than Group 1, and Group 3 will have fewer risky driving events than Group 2, using a one-way ANOVA and adjusting for two-sided multiple comparisons. Based on these sample sizes and less than 10% loss to follow-up, we will recruit and enroll 80 dyads per group (a total of 240).

For the primary outcome of risky driving events (hard braking, sudden acceleration, speeding), the overall rate and rate for each type of event per 1,000 miles driven will be calculated for each teen at 6-month follow-up. To compare the differences among the 3 groups, one-way ANOVAs will be used. To model the effects of the intervention on the rates of risky driving events per 1,000 miles driven, a Quasi-Poisson analysis for over-dispersed count data will be conducted, where 2 dummy variables indicating Groups 2 and 3 will be created as covariates to determine risk ratios of risky driving events among the 3 groups, adjusting for the other potential covariates.

For the primary outcome of unsafe driving behaviors (speeding, distracted driving, no seatbelt use), to model the effects of the intervention on the proportion of unsafe behaviors per 1,000 miles driven, Quasi-Poisson analysis for over-dispersed count data will be implemented, where two dummy variables indicating Groups 2 and 3 will be created as covariates to determine the risk ratios of unsafe driving behavior among the 3 groups, adjusting for the potential covariates described above. In addition, a secondary analysis of correlation coefficients will be conducted to ascertain if and how the risky driving events and unsafe driving behaviors are correlated and whether the correlations are different between the study groups. The potential covariates will be adjusted.

For the secondary outcome of frequency and quality of parent-teen communication about driving safety, to model the effects of the intervention on differences in frequency and quality of parent-teen communication among the 3 groups, an Analysis of covariance (ANCOVA) will be used for each outcome, adjusting for the potential covariates. In addition, the following secondary analyses will be performed: 1) Correlation analysis will be conducted to examine if and how frequency and quality of parent-teen communication are correlated; 2) Longitudinal data analyses using mixed-effect models will be performed to further ascertain if the parent training enhances parent-teen communication over time with a focus on how these measures change over time in Group 3; 3) We will conduct further analysis on risky driving events and unsafe driving behaviors using the Quasi-Poisson analysis by including the measures of frequency and quality of parent-teen communication as three additional covariates to evaluate the effects of parent-teen communication, adjusting for group differences.

For the secondary outcome of recidivism, a Cox Proportional Hazards Model will be established to ascertain whether and how much the Device Feedback and/or Device Feedback plus Parent Training intervention reduce the hazard of teen recidivism, adjusting for the potential covariates described above. In addition, the measures of risky driving events and unsafe driving behaviors will be specifically included in the Cox Proportional Hazards Models to examine how risky driving events and unsafe driving behaviors increase the hazards of recidivism, adjusting for potential covariates.

17.2 Describe the steps that will be taken to secure the data (e.g., training, authorization of access, password protection, encryption, physical controls, certificates of confidentiality, and separation of identifiers and data) during storage, use, and transmission.

A unique participant ID will be created for each subject. The list linking the assigned ID to the individual subject will be maintained separately from the other research data. All data files used for analysis will only have the ID and will not contain any other personal information. Data will be stored on secure, password protected computers.

17.3 Describe any procedures that will be used for quality control of collected data.

N/A

17.4 Describe how data or specimens will be handled study-wide:

17.4.1 What information will be included in that data or associated with the specimens?

Participant data will have unique participant IDs that are stored on a secure password protected computer.

17.4.2 Where and how data or specimens will be stored?
Until completion of the study.

17.4.3 How long the data or specimens will be stored?
Until completion of the study.

17.4.4 Who will have access to the data or specimens?
Research team members.

17.4.5 Who is responsible for receipt or transmission of the data or specimens?
Research team members.

17.4.6 How data or specimens will be transported?
By research team members.

18.0 Provisions to Protect the Privacy Interests of Subjects

18.1 Describe the steps that will be taken to protect subjects' privacy interests. "Privacy interest" refers to a person's desire to place limits on whom they interact or whom they provide personal information.
A unique participant ID will be created for each subject. The list linking the assigned ID to the individual subject will be maintained separately from the other research data on password protected computers. All data files used for analysis will only have the ID and will not contain any other personal information.

18.2 Describe what steps you will take to make the subjects feel at ease with the research situation in terms of the questions being asked and the procedures being performed. "At ease" does not refer to physical discomfort, but the sense of intrusiveness a subject might experience in response to questions, examinations, and procedures.
All participants will be given a phone number that connects them to a research team member. Participants can call and ask research related questions at any time (24/7).

18.3 Indicate how the research team is permitted to access any sources of information about the subjects.
Our research team will not access any source of personal information about the subjects.

19.0 Economic Burden to Subjects

19.1 Describe any costs that subjects may be responsible for because of participation in the research.
All costs related to the research parts of this study will be covered by the research team. However, the parts of the study that would be done for routine clinical care will be billed to an insurance company or third party payer. Further, if a participant were to be involved in a crash while participating in this study, participants are responsible for any costs associated with damages. Participants may have to pay any costs that the insurance company or third party payer does not pay. There may be additional costs related to travel during this study. We will not provide money to help with these costs. A parking voucher will be provided if participants come to NCH for a study visit. This reimbursement may be taxable. The study team will discuss this with participants.

20.0 Consent Process

20.1 Indicate whether you will you be obtaining consent, and if so describe:

20.1.1 Where will the consent process take place
In-person: Consent will take place at various Ohio Juvenile Traffic Courts in a private room near the courtroom or at location convenient for the participants (i.e., participant home, school, library).
Virtual: Consent will take place over video conference with the researcher, teen, and parent present. The consent will be signed through REDCap.

20.1.2 Any waiting period available between informing the prospective subject and obtaining the consent.
If participants want time to consider participation, research team members will give them time for consideration.

20.1.3 Any process to ensure ongoing consent.
N/A

20.1.4 Whether you will be following "SOP: Informed Consent Process for Research (HRP-090)." (Yes)

Subjects who are not yet adults (infants, children, teenagers)

20.1.5 Describe the criteria that will be used to determine whether a prospective subject has not attained the legal age for consent to treatments or procedures involved in the research under the applicable law of the jurisdiction in which the research will be conducted. (E.g., individuals under the age of 18 years.)

Participants will be asked their age, and their age will be confirmed by a legal guardian.

20.1.6 Describe whether parental permission will be obtained from:

One parent even if the other parent is alive, known, competent, reasonably available, and shares legal responsibility for the care and custody of the child.

20.2 Indicate whether assent will be obtained from all, some, or none of the children. If assent will be obtained from some children, indicate which children will be required to assent. When assent of children is obtained describe whether and how it will be documented.

Written assent of the child will be obtained and documented at the same time written consent from a parent/legal guardian is obtained.

21.0 Process to Document Consent in Writing

21.1 Describe whether you will be following "SOP: Written Documentation of Consent (HRP-091)." If not, describe whether and how consent of the subject will be documented in writing.

Yes, we will collect written documentation of consent.

22.0 Setting

22.1 Describe the sites or locations where your research team will conduct the research.

22.1.1 Identify where your research team will identify and recruit potential subjects.

In-person: Identification and recruitment of individuals will occur at various Juvenile Traffic Courts in Ohio such as the Franklin County Juvenile Traffic Court and at different venues such as Carteens.
Virtual: Potential participants will have the ability to scan a QR code at the various Juvenile Traffic Courts and different local Franklin County Juvenile Traffic Court in Columbus, Ohio and through multiple online avenues including social media, research databases (e.g., Studysearch, ResearchMatch), and other relevant listservs (e.g. Peachjar).

22.1.2 Identify where research procedures will be performed.

Research procedures will occur at the courthouse and at the participant's home (or a location that is convenient to them based on their personal preference).

22.1.3 Describe the composition and involvement of any community advisory board.

N/A

23.0 Resources Available

23.1 Describe the resources available to conduct the research: For example, as appropriate:

23.1.1 Justify the feasibility of recruiting the required number of suitable subjects within the agreed recruitment period. For example, how many potential subjects do you have access to? What percentage of those potential subjects do you need to recruit?

With our previous pilot study, our research team demonstrated success recruiting from the courthouse. Specifically, for our pilot study we were able to enroll 10 parent-teen dyads within one week. Based on existing traffic citation and court disposition data, the number of teens 16- or 17-years who committed a traffic violation(s) was 2,843 in 2016 and 2,847 in 2017, 13% of whom had a previous offense. These numbers provide an ample base population to enroll the targeted sample of 240 parent-teen dyads. Enrolled teens who sustain an additional violation can continue their participation in the study as long as they still meet the eligibility criteria.

23.1.2 Describe the availability of medical or psychological resources that subjects might need as a result of an anticipated consequences of the human research.
NCH has medical and psychological resources that are noted in our study consent form for participants to access, if needed.

NCH has medical and psychological resources that are noted in our study consent form for participants to access, if needed.

23.1.3 Describe your process to ensure that all persons assisting with the research are adequately informed about the protocol, the research procedures, and their duties and functions.

All research staff have been trained on research procedures and are adequately prepared to perform all research related tasks as needed.

24.0 Protected Health Information Recording

24.1 Indicate which subject identifiers will be recorded for this research.

☒ Name
☒ Complete Address
☒ Telephone or Fax Number
☐ Social Security Number (do not check if only used for ClinCard)
☒ Dates (treatment dates, birth date, date of death)
☒ Email address, IP address or url
☐ Medical Record Number or other account number
☐ Health Plan Beneficiary Identification Number
☒ Full face photographic images and/or any comparable images (x-rays)
☐ Account Numbers
☒ Certificate/License Numbers
☒ Vehicle Identifiers and Serial Numbers (e.g. VINs, License Plate Numbers)
☐ Device Identifiers and Serial Numbers
☐ Biometric identifiers, including finger and voice prints
☐ Other number, characteristic or code that could be used to identify an individual
☐ None (Complete De-identification Certification Form)

24.2 Check the appropriate category and attach the required form* on the Local Site Documents, #3. Other Documents, page of the application. (Choose one.)

☒ Patient Authorization will be obtained. (Include the appropriate HIPAA language (see Section 14 of consent template) in the consent form OR attach the HRP-900, HIPAA AUTHORIZATION form.)
☐ Protocol meets the criteria for waiver of authorization. (Attach the HRP-901, WAIVER OF HIPAA AUTHORIZATION REQUEST form.)
☐ Protocol is using de-identified information. (Attach the HRP-902, DE-IDENTIFICATION CERTIFICATION form.) (Checked "None" in 1.0 above)
☐ Protocol involves research on decedents. (Attach the HRP-903, RESEARCH ON DECEDENTS REQUEST form.)
☐ Protocol is using a limited data set and data use agreement. (Contact the Office of Technology Commercialization to initiate a Limited Data Use Agreement.

***Find the HIPAA forms in the IRB Website Library, Templates.**

Attach the appropriate HIPAA form on the “Local Site Documents, #3. Other Documents”, page of the application.

24.3 How long will identifying information on each participant be maintained?

Identifying information will be kept until the end of the study.

24.4 Describe any plans to code identifiable information collected about each participant.

Pseudonyms for each teen and parent will be created. The list linking the assigned pseudonym and email will be maintained separately from other research data. All files used for analysis will only have the pseudonym and will not contain any other personal information.

24.5 Check each box that describes steps that will be taken to safeguard the confidentiality of information collected for this research:

☒ **Research records will be stored in a locked cabinet in a secure location**
☒ **Research records will be stored in a password-protected computer file**
☒ **The list linking the assigned code number to the individual subject will be maintained separately from the other research data**
☒ **Only certified research personnel will be given access to identifiable subject information**

24.6 Describe the provisions included in the protocol to protect the privacy interests of subjects, where "privacy interests" refer to the interest of individuals in being left alone, limiting access to them, and limiting access to their information. (This is not the same provision to maintain the confidentiality of data.)

All data collected will be stored on a password protected computer that can only be accessed by our research team. Analysis will utilize aggregated data only.

25.0 Confidential Health Information

25.1 Please mark all categories that reflect the nature of health information to be accessed and used as part of this research.

☒ Demographics (age, sex, educational level)
☐ Diagnosis
☐ Laboratory reports
☐ Radiology reports
☐ Discharge summaries
☐ Procedures/Treatments received
☐ Dates related to course of treatment (admission, surgery, discharge)
☐ Billing information
☐ Names of drugs and/or devices used as part of treatment
☐ Location of treatment
☐ Name of treatment provider
☐ Surgical reports
☐ Other information related to course of treatment
☐ None

25.2 Please discuss why it is necessary to access and review the health information noted in your response above.

It is necessary to collect participant demographics as part of our inclusion criteria for the study.

25.3 Is the health information to be accessed and reviewed the minimal necessary to achieve the goals of this research?

☒ Yes ☐ No

25.4 Will it be necessary to record information of a sensitive nature? ☐ Yes ☒ No

25.5 Do you plan to obtain a federally-issued Certificate of Confidentiality as a means of protecting the confidentiality of the information collected? ☐ Yes ☒ No
