# Supplementary material for: In-Vehicle Feedback With or Without Parent Communication Training and Teenage Driving Behaviors: A Randomized Clinical Trial
Source: JAMA Netw Open. 2026 Apr 24;9(4):e268631. doi: 10.1001/jamanetworkopen.2026.8631 (PMC13109799; doi:10.1001/jamanetworkopen.2026.8631)
Supplement: Supplement 2. — eTable 1. Proportion of Miles Driven With Detected Seatbelt Nonuse (n = 83) eTable 2. Self-Reported Distracted Driving Behaviors During the Past Two Weeks eTable 3. Incidence Rate Ratios (Unadjusted and Adjusted) of Specific Risky Driving Events per 1,000 Miles Driven Across Study Arms (n = 160,095 Trips by 240 Teens) eTable 4. Proportion Ratios (Unadjusted and Adjusted) of Miles Driven Involving Specific Speeding Behaviors Across Study Arms (n = 160,095 Trips by 240 Teens) eTable 5. Odds Ratios (Unadjusted and Adjusted) of Risky Driving Events Across Study Arms (n = 160,095 Trips by 240 Teens) eTable 6. Odds Ratios (Unadjusted and Adjusted) of Miles Driven Involving Speeding Behaviors Across Study Arms (n = 160,095 Trips by 240 Teens) eTable 7. Odds Ratios (Unadjusted and Adjusted) of Specific Risky Driving Events Across Study Arms (n = 160,095 Trips by 240 Teens) eTable 8. Odds Ratios (Unadjusted and Adjusted) of Miles Driven Involving Specific Speeding Behaviors Across Study Arms (n = 160,095 Trips by 240 Teens) [file jamanetwopen-e268631-s002.pdf]

Supplementary Online Content

Yang J, Zhang Y, Alshaikh E, et al. In-vehicle feedback with or without parent communication training and teenage driving behaviors: a randomized clinical trial. *JAMA Netw Open.* 2026;9(4):e268631. doi:10.1001/jamanetworkopen.2026.8631

eTable 1. Proportion of Miles Driven With Detected Seatbelt Nonuse (n = 83)

eTable 2. Self-Reported Distracted Driving Behaviors During the Past Two Weeks

eTable 3. Incidence Rate Ratios (Unadjusted and Adjusted) of Specific Risky Driving Events per 1,000 Miles Driven Across Study Arms (n = 160,095 Trips by 240 Teens)

eTable 4. Proportion Ratios (Unadjusted and Adjusted) of Miles Driven Involving Specific Speeding Behaviors Across Study Arms (n = 160,095 Trips by 240 Teens)

eTable 5. Odds Ratios (Unadjusted and Adjusted) of Risky Driving Events Across Study Arms (n = 160,095 Trips by 240 Teens)

eTable 6. Odds Ratios (Unadjusted and Adjusted) of Miles Driven Involving Speeding Behaviors Across Study Arms (n = 160,095 Trips by 240 Teens)

eTable 7. Odds Ratios (Unadjusted and Adjusted) of Specific Risky Driving Events Across Study Arms (n = 160,095 Trips by 240 Teens)

eTable 8. Odds Ratios (Unadjusted and Adjusted) of Miles Driven Involving Specific Speeding Behaviors Across Study Arms (n = 160,095 Trips by 240 Teens)

This supplementary material has been provided by the authors to give readers additional information about their work.

© 2026 Yang J et al. *JAMA Network Open.*

**eTable 1.** Proportion of Miles Driven With Detected Seatbelt Nonuse (n = 83)				
	**Overall (n=83)**	**Control (n=30)**	**Driving Feedback Only (n=24)**	**Driving Feedback plus Parent Training (n=29)**
Participants with vehicles capable of detecting seatbelt nonuse, n (%)	83 (100)	30 (36)	24 (29)	29 (35)
Participants with at least one detected seatbelt nonuse, n (%)[a]	49 (59)	17 (57)	14 (58)	18 (62)
Trips made by vehicles capable of detecting seatbelt nonuse, n (%)	56,478 (100)	19,879 (35)	15,265 (27)	21,334 (38)
Trips with at least one detected seatbelt nonuse, n (%)	8,571 (15.2)	2,818 (14.2)	2792 (18.2)	2,961(13.9)
Mean miles driven per trip with detected seatbelt nonuse (SD)	5.5 (8.5)	5.5 (9.3)	5.0 (6.9)	5.9 (9.0)
Proportion of miles driven with detected seatbelt nonuse, mean (SD)	0.09 (0.3)	0.09 (0.3)	0.12 (0.3)	0.07 (0.2)

[a]Denominator is the number of participants with vehicles capable of detecting seatbelt nonuse

© 2026 Yang J et al. *JAMA Network Open.*

eTable 2. Self-Reported Distracted Driving Behaviors During the Past Two Weeks

Distracted Driving Behaviors[b]	Overall (n=229)[a]	Control (n=76)	Driving Feedback Only (n=74)	Driving Feedback plus Parent Training (n=79)
**Talk on a hands-free device**				
Never	31.3	29.4	29.8	34.7
Less than half of the time	56.3	54.6	56.8	57.4
Half the time or more	7.8	9.8	8.2	5.4
No answer	4.6	6.2	5.2	2.5
**Talk on a hand-held phone**				
Never	55.6	51.4	52.4	63.1
Less than half of the time	37.3	40.0	38.0	34.0
Half the time or more	2.2	2.0	4.2	0.5
No answer	4.8	6.6	5.4	2.5
**Send text messages**				
Never	47.7	46.6	41.7	54.9
Less than half of the time	43.5	45.3	45.9	39.4
Half the time or more	3.9	1.8	6.6	3.2
No answer	4.9	6.3	5.8	2.5
**Read text messages**				
Never	38.6	34.0	36.7	45.1
Less than half of the time	51.7	56.3	50.7	48.3
Half the time or more	4.9	3.6	7.0	4.2
No answer	4.7	6.2	5.5	2.5
**View a social media app**				
Never	67.7	67.1	62.7	73.4
Less than half of the time	24.3	24.7	26.6	21.5
Half the time or more	3.5	2.1	5.6	2.8
No answer	4.5	6.1	5.1	2.4
**Post to a social media app**				
Never	84.0	82.1	80.9	88.9
Less than half of the time	10.6	11.8	11.9	8.3
Half the time or more	0.8	0.0	2.0	0.5
No answer	4.5	6.1	5.2	2.4
**Composite frequency[c]**				
Never	54.2	51.8	50.7	60.0
Less than half of the time	37.3	38.8	38.3	34.8
Half the time or more	3.9	3.2	5.6	2.7
No answer	4.7	6.2	5.4	2.4

[a]Of the 240 teens, 229 (95.4%) completed at least one biweekly survey during the six-month study period.

© 2026 Yang J et al. *JAMA Network Open.*

[b]Participants were asked how frequently they engaged in each of six distracted driving behaviors during the past two weeks. For each participant, responses were standardized by the total number of surveys completed.
[c]Represents the mean percentage across the six distracted driving behaviors.

© 2026 Yang J et al. *JAMA Network Open.*

**eTable 3.** Incidence Rate Ratios (Unadjusted and Adjusted) of Specific Risky Driving Events per 1,000 Miles Driven Across Study Arms (n = 160,095 Trips by 240 Teens)				
	**Unadjusted Incidence Rate Ratio (IRR),[a] (95%CI)**		**Adjusted Incidence Rate Ratio (aIRR),[a,b] (95%CI)**	
**Specific Kinematic Events**				
**Hard Braking[c]**				
Driving Feedback Only vs. Control	0.96	(0.73-1.25)	0.94	(0.72-1.23)
Driving Feedback plus Parent Training vs. Control	0.75	(0.57-0.98)	0.77	(0.58-1.01)
Driving Feedback plus Parent Training vs. Driving Feedback Only	0.78	(0.60-1.03)	0.82	(0.62-1.08)
Sex (Male vs. Female)			1.39	(1.11-1.73)
Age (17 vs. 16, y)			0.85	(0.68-1.06)
**Sudden Acceleration[d]**				
Driving Feedback Only vs. Control	1.20	(0.73-2.00)	1.23	(0.75-2.01)
Driving Feedback plus Parent Training vs. Control	1.03	(0.62-1.71)	1.07	(0.65-1.74)
Driving Feedback plus Parent Training vs. Driving Feedback Only	0.85	(0.52-1.43)	0.87	(0.53-1.45)
Sex (Male vs. Female)			2.13	(1.42-3.19)
Age (17 vs. 16, y)			1.10	(0.74-1.65)
**Specific Speeding Events**				
**Speed >10 mph Over the Posted Speed Limit**				
Driving Feedback Only vs. Control	0.99	(0.75-1.3)	1.11	(0.85-1.46)
Driving Feedback plus Parent Training vs. Control	0.88	(0.67-1.16)	0.99	(0.76-1.30)
Driving Feedback plus Parent Training vs. Driving Feedback Only	0.89	(0.68-1.18)	0.89	(0.68-1.18)
Sex (Male vs. Female)			1.07	(0.86-1.34)
Age (17 vs. 16, y)			1.08	(0.87-1.35)
**Speed >75 mph**				
Driving Feedback Only vs. Control	1.16	(0.76-1.77)	1.16	(0.77-1.74)
Driving Feedback plus Parent Training vs. Control	0.82	(0.54-1.25)	0.81	(0.54-1.22)
Driving Feedback plus Parent Training vs. Driving Feedback Only	0.70	(0.46-1.08)	0.70	(0.47-1.05)
Sex (Male vs. Female)			1.27	(0.91-1.78)
Age (17 vs. 16, y)			1.35	(0.96-1.89)

IRR: Incidence Rate Ratio; **aIRR:** Adjusted Incidence Rate Ratio; **CI:** Confidence Interval; **mph**: Miles per Hour.

[a] IRR was estimated using the trip-based mixed-effects zero-inflated negative binomial regression model's count component, with miles driven as an

[b] Adjusting for teens' sex and age.

[c] ≤ -0.45 g-force.

[d] > 0.35 g-force.

© 2026 Yang J et al. *JAMA Network Open.*

eTable 4. Proportion Ratios (Unadjusted and Adjusted) of Miles Driven Involving Specific Speeding Behaviors Across Study Arms (n = 160,095 Trips by 240 Teens)

	Unadjusted Proportion Ratio (Exponentiated β coefficients [expβ],[a] (95%CI)		Adjusted Proportion Ratio (aexpβ),[a,b] (95%CI)	
**Miles Driven Involving Speeding Behaviors**				
**Speed >10 mph Over the Posted Speed Limit**				
Driving Feedback Only vs. Control	0.98	(0.83-1.19)	0.98	(0.83-1.19)
Driving Feedback plus Parent Training vs. Control	0.95	(0.81-1.15)	0.95	(0.81-1.15)
Driving Feedback plus Parent Training vs. Driving Feedback Only	0.97	(0.83-1.18)	0.97	(0.83-1.18)
Sex (Male vs. Female)			1.06	(0.93-1.24)
Age (17 vs. 16, y)			0.97	(0.85-1.13)
**Speed >75 mph**				
Driving Feedback Only vs. Control	0.80	(0.67-0.99)	0.79	(0.67-0.98)
Driving Feedback plus Parent Training vs. Control	0.78	(0.65-0.96)	0.78	(0.65-0.96)
Driving Feedback plus Parent Training vs. Driving Feedback Only	0.97	(0.82-1.20)	0.98	(0.82-1.21)
Sex (Male vs. Female)			1.08	(0.94-1.28)
Age (17 vs. 16, y)			1.12	(0.83-1.21)

expβ: Exponentiated β coefficients (multiplicative effects on the mean proportion); **aexpβ**: Adjusted Exponentiated β coefficients; **CI:** Confidence Interval; **mph**: Miles per Hour.

[a] expβ was estimated using the trip-based mixed-effects zero-inflated Beta regression model's proportion component.

[b] Adjusting for teens' sex and age.

© 2026 Yang J et al. *JAMA Network Open.*

eTable 5. Odds Ratios (Unadjusted and Adjusted) of Risky Driving Events Across Study Arms (n = 160,095 Trips by 240 Teens)

Risky Driving Events	Unadjusted Odds Ratio (OR),[a] (97.5%CI)		p-value	Adjusted Odds Ratio (aOR),[a,b] (97.5%CI)		p-value
Driving Feedback Only vs. Control	0.80	(0.50-1.28)	< 0.01[c]	0.79	(0.52-1.20)	< 0.01[c]
Driving Feedback plus Parent Training vs. Control	3.29	(2.32-4.67)		2.51	(1.81-3.49)	
Driving Feedback plus Parent Training vs. Driving Feedback Only	4.17	(2.84-6.12)		3.23	(2.23-4.67)	
Sex (Male vs. Female)				0.85	(0.54-1.12)	
Age (17 vs. 16, y)				1.70	(1.29-2.2.25	

OR: Odds Ratio; **aOR**: Adjusted Odds Ratio; **CI:** Confidence Interval.

[a]OR was estimated using the trip-based mixed-effects zero-inflated negative binomial model's zero component.

[b]Adjusting for teens' sex and age.

[c]The p-value was calculated using a Chi-square test comparing differences across the three arms. A significance threshold of $p < 0.025$ was used, based on a Bonferroni adjustment for two pre-specified co-primary endpoints. The corresponding confidence intervals were also Bonferroni-adjusted.

© 2026 Yang J et al. *JAMA Network Open.*

eTable 6. Odds Ratios (Unadjusted and Adjusted) of Miles Driven Involving Speeding Behaviors Across Study Arms (n = 160,095 Trips by 240 Teens)

Miles Driven Involving Speeding Behaviors	Unadjusted Odds Ratio (OR),[a] (97.5%CI)		p-value	Adjusted Odds Ratio (aOR),[a,b] (97.5%CI)		p-value
Driving Feedback Only vs. Control	0.85	(0.85-0.90)	< 0.01[c]	0.86	(0.84-0.89)	< 0.01[c]
Driving Feedback plus Parent Training vs. Control	0.96	(0.96-1.02)		0.99	(0.97-1.03)	
Driving Feedback plus Parent Training vs. Driving Feedback Only	1.12	(1.10-1.17)		1.15	(1.12-1.19)	
Sex (Male vs. Female)				0.78	(0.76-0.79)	
Age (17 vs. 16, y)				1.08	(1.05-1.11)	

OR: Odds Ratio; **aOR**: Adjusted Odds Ratio; **CI:** Confidence Interval.
[a]OR was estimated using the trip-based mixed-effects zero-inflated beta model's zero component.
[b]Adjusting for teens' sex and age.
[c]The p-value was calculated using a Chi-square test comparing differences across the three arms. A significance threshold of $p < 0.025$ was used, based on a Bonferroni adjustment for two pre-specified co-primary endpoints. The corresponding confidence intervals were also Bonferroni-adjusted.

© 2026 Yang J et al. *JAMA Network Open.*

**eTable 7.** Odds Ratios (Unadjusted and Adjusted) of Specific Risky Driving Events Across Study Arms (n = 160,095 Trips by 240 Teens)				
**Specific Risky Driving Events**	**Unadjusted Odds Ratio (OR),[a] (95%CI)**		**Adjusted Odds Ratio (aOR),[a,b] (95%CI)**	
**Specific Kinematic Events**				
**Hard Braking[d]**				
Driving Feedback Only vs. Control	0.82	(0.63-1.06)	0.67	(0.50-0.90)
Driving Feedback plus Parent Training vs. Control	1.70	(1.34-2.16)	1.93	(1.41-2.65)
Driving Feedback plus Parent Training vs. Driving Feedback Only	2.08	(1.61-2.70)	2.86	(1.96-4.35)
Sex (Male vs. Female)			1.76	(1.27-2.43)
Age (17 vs. 16, y)			0.74	(0.53-1.03)
**Sudden Acceleration[e]**				
Driving Feedback Only vs. Control	1.51	(1.01-2.26)	1.77	(1.18-2.65)
Driving Feedback plus Parent Training vs. Control	3.19	(2.17-4.70)	3.63	(2.48-5.31)
Driving Feedback plus Parent Training vs. Driving Feedback Only	2.13	(1.61-2.78)	2.04	(1.56-2.70)
Sex (Male vs. Female)			1.02	(0.67-1.55)
Age (17 vs. 16, y)			1.63	(1.25-2.11)
**Specific Speeding Events**				
**Speed >10 mph Over the Posted Speed Limit**				
Driving Feedback Only vs. Control	0.75	(0.57-0.98)	0.58	(0.44-0.76)
Driving Feedback plus Parent Training vs. Control	0.81	(0.62-1.04)	0.61	(0.47-0.78)
Driving Feedback plus Parent Training vs. Driving Feedback Only	1.08	(0.83-1.39)	1.05	(0.80-1.37)
Sex (Male vs. Female)			0.70	(0.56-0.87)
Age (17 vs. 16, y)			2.17	(1.72-2.74)
**Speed >75 mph**				
Driving Feedback Only vs. Control	0.84	(0.79-0.89)	0.84	(0.79-0.89)
Driving Feedback plus Parent Training vs. Control	1.15	(1.08-1.23)	1.17	(1.09-1.25)
Driving Feedback plus Parent Training vs. Driving Feedback Only	1.37	(1.28-1.47)	1.39	(1.32-1.49)
Sex (Male vs. Female)			0.84	(0.80-0.89)
Age (17 vs. 16, y)			0.88	(0.83-0.93)

OR: Odds Ratio; **aOR**: Adjusted Odds Ratio; **CI:** Confidence Interval; **mph:** Miles per Hour.
[a]OR was estimated using the trip-based mixed-effects zero-inflated negative binomial model's zero component.
[c]Adjusting for teens' sex and age.
[d]≤ -0.45 g-force.

© 2026 Yang J et al. *JAMA Network Open.*

[e]> 0.35 g-force.

© 2026 Yang J et al. *JAMA Network Open.*

eTable 8. Odds Ratios (Unadjusted and Adjusted) of Miles Driven Involving Specific Speeding Behaviors Across Study Arms (n = 160,095 Trips by 240 Teens)

	Unadjusted Odds Ratio (OR),[a] (95%CI)		Adjusted Odds Ratio (aOR),[a,b] (95%CI)	
**Miles Driven Involving Specific Speeding Behaviors**				
**Speed >10 mph Over the Posted Speed Limit**				
Driving Feedback Only vs. Control	0.78	(0.75-0.80)	0.77	(0.75-0.79)
Driving Feedback plus Parent Training vs. Control	0.85	(0.83-0.88)	0.86	(0.84-0.89)
Driving Feedback plus Parent Training vs. Driving Feedback Only	1.11	(1.08-1.14)	1.12	(1.10-1.15)
Sex (Male vs. Female)			0.85	(0.83-0.87)
Age (17 vs. 16, y)			1.01	(0.98-1.03)
**Speed >75 mph**				
Driving Feedback Only vs. Control	0.73	(0.70-0.76)	0.72	(0.69-0.76)
Driving Feedback plus Parent Training vs. Control	1.18	(1.13-1.24)	1.20	(1.14-1.26)
Driving Feedback plus Parent Training vs. Driving Feedback Only	1.62	(1.55-1.70)	1.66	(1.58-1.73)
Sex (Male vs. Female)			0.81	(0.78-0.84)
Age (17 vs. 16, y)			0.85	(0.82-0.89)

OR: Odds Ratio; **aOR**: Adjusted Odds Ratio; **CI:** Confidence Interval; **mph:** Miles per Hour.
[a]OR was estimated using the trip-based mixed-effects zero-inflated beta model's zero component.
[b]Adjusting for teens' sex and age.

© 2026 Yang J et al. *JAMA Network Open.*
