# Supplementary material for: In-Vehicle Feedback With or Without Parent Communication Training and Teenage Driving Behaviors: A Randomized Clinical Trial
Source: JAMA Netw Open. 2026 Apr 24;9(4):e268631. doi: 10.1001/jamanetworkopen.2026.8631 (PMC13109799; doi:10.1001/jamanetworkopen.2026.8631)
Supplement: Supplement 3. — Data Sharing Statement [file jamanetwopen-e268631-s003.pdf]

Data Sharing Statement

Yang. In-Vehicle Feedback With or Without Parent Communication Training and Teenage Driving Behaviors. *JAMA Netw Open*. Published April 24, 2026.
doi:10.1001/jamanetworkopen.2026.8631

Data
Additional Information: The study was registered on ClinicalTrials.gov Registry (NCT04317664), (https://clinicaltrials.gov/study/NCT04317664).
Data available: Yes
Data types: Deidentified participant data, Data dictionary
How to access data: The datasets used and/or analyzed during the current study are available from the corresponding author upon reasonable request. The corresponding author had full access to all the data in the study and takes responsibility for the integrity of the data and the accuracy of the data analysis. The corresponding author will be responsible for providing access to research data requested by third parties as freely and timely as possible unless a legal obligation restricts access to the data (e.g., non-disclosure agreement), intellectual property protection, ethical approval requirements, ethical or security reasons, or other legitimate reasons.
When available: With publication

Supporting Documents
Document types: Informed consent form
How to access documents: The datasets used and/or analyzed during the current study are available from the corresponding author upon reasonable request. The corresponding author had full access to all the data in the study and takes responsibility for the integrity of the data and the accuracy of the data analysis. The corresponding author will be responsible for providing access to research data requested by third parties as freely and timely as possible unless a legal obligation restricts access to the data (e.g., non-disclosure agreement), intellectual property protection, ethical approval requirements, ethical or security reasons, or other legitimate reasons.
When available: With publication

Additional Information
Who can access the data: The corresponding author will be responsible for providing access to research data requested by third parties as freely and timely as possible.
Types of analyses: Data used for this analysis
Mechanisms of data availability: Data will be made available after approval of a proposal and with a signed data access agreement, without investigator support.
